# Maternal and child nutrition in the Lives Saved Tool: Results of a recent update

**DOI:** 10.7189/jogh.12.08005

**Published:** 2022-12-30

**Authors:** Hannah Tong, Ellen Piwoz, Marie T Ruel, Kenneth H Brown, Robert E Black, Neff Walker

**Affiliations:** 1 Department of International Health, Bloomberg School of Public Health, Johns Hopkins University, Baltimore, Maryland, USA; 2 Independent Consultant, Annapolis, Maryland, USA; 3 Poverty, Health, and Nutrition Division, International Food Policy Research Institute, Washington, District of Columbia, USA; 4 Department of Nutrition and Institute for Global Nutrition, University of California, Davis, California, USA

## Abstract

**Background:**

The Lives Saved Tool (LiST) is a mathematical modelling tool for estimating the survival, health, and nutritional impacts of scaling intervention coverage in low- and middle-income countries (LMICs). Various nutrition interventions are included in LiST and are regularly (and independently) reviewed and updated as new data emerge. This manuscript describes our latest in-depth review of nutrition evidence, focusing on intervention efficacy, appropriate population-affected fractions, and new interventions for potential inclusion in the LiST model.

**Methods:**

An external advisory group (EAG) was assembled to review evidence from systematic reviews on intervention-outcome (I-O) pairs for women and children under five years of age. GRADE quality was assigned to each pair based on a LiST-specific checklist to facilitate consistent decisions during the consideration. For existing interventions with new information, the EAG was asked to recommend whether to update the default efficacy values and population-affected fractions. For the new interventions, the EAG decided whether there was sufficient evidence of benefit, and in affirmative cases, information on the efficacy and affected fraction values that could be used. Decisions were based on expert group consensus.

**Results:**

Overall, the group reviewed 53 nutrition-related I-O pairs, including 25 existing and 28 new ones. Efficacy and population-affected fractions were updated for seven I-O pairs; three pairs were updated for efficacy estimates only, three were updated for population-affected fractions only; and nine new I-O pairs were added to the model, bringing the total of nutrition-related I-O pairs to 34. Included in the new I-O pairs were two new nutrition interventions added to LIST: zinc fortification and neonatal vitamin A supplementation.

**Conclusions:**

For modelling tools like LiST to be useful, it is crucial to update interventions, efficacy and population-affected fractions as new evidence becomes available. The present updates will enable LiST users to better estimate the potential health, nutrition, and survival benefits of investing in nutrition.

The Lives Saved Tool (LiST) is a mathematical model that estimates the impact of changes in coverage of selected interventions on health outcomes among pregnant women and children less than five years of age. The model is built around an assumption that changes in coverage of health and nutrition interventions drive health outcomes, either directly or by modifying specific risk factors for these outcomes [1].

LiST modelling starts with describing the health status of women and children in a base year for all low- and middle-income countries (LMICs) identified by the World Bank. This description includes levels and causes of mortality (maternal, neonatal, and in 1-59-month-old children), risk factors for mortality or other adverse health outcomes (eg, nutrient deficiencies, age-specific levels of stunting and wasting, birth status (including pre-term and small-for-gestational-age) breastfeeding practices) and base year levels of coverage of efficacious nutrition and health interventions that have a known impact on maternal and child health.

Each intervention in the LiST model is selected based on evidence of efficacy in reducing the risk of cause-specific mortality or the prevalence of a risk factor of mortality or morbidity. LiST can simulate a hypothetical increase in the coverage of one or more interventions and estimate related changes in the prevalence of risk factors and rates of mortality based on the changes in coverage of each intervention and its efficacy on the segment of the population who is likely to benefit from it. It also incorporates demographic changes in the population based on projected future fertility and mortality rates. For more details on the model visit the LiST website [2].

LiST has been used by many organizations, including academic institutions, non-government organizations, and government ministries, typically for strategic planning, budgeting, evaluation, or advocacy [3], but most often to model the global effects of scaling up interventions on maternal and child health and nutritional status outcomes [4-7].

LiST primarily relies on information from systematic reviews for estimates of efficacy because they include evidence from multiple studies, often from diverse LMICs, and therefore provide more robust estimates of efficacy than individual studies. However, these analyses may not produce the specific values that are needed in LiST.

Key issues for maintaining and updating the LiST model are adding new interventions as new evidence on efficacy becomes available and updating estimates of efficacy for existing interventions. A critical issue for nutrition interventions is the population to which the benefits of the intervention will accrue. In the model, this subset of the population is referred to as the affected fraction, which may be defined as the percentage of the population that has an insufficient intake of a nutrient in their diet or the prevalence of a particular deficiency according to a recognized biomarker of nutritional status (eg, prevalence of a low concentration of serum retinol). However, most nutrition intervention trials do not screen for pre-existing deficiency; rather, they are conducted in areas with high poverty levels, poor dietary diversity, or high food insufficiency, with the assumption that most of the population would benefit from the intervention. Therefore, a critical issue for LiST is selecting the appropriate measure of the affected fraction and matching it to the appropriate intervention efficacy value.

The last review and expansion of the nutrition components of LiST was conducted in 2017 [8]. Since then, more evidence on efficacy of different nutrition interventions has become available. This study aims to summarize the 2021-22 review process and decisions made regarding LiST inputs. The review examined data on nutrition intervention efficacy, including previously included and new interventions and affected fractions.

## METHODS

**Figure 1:**
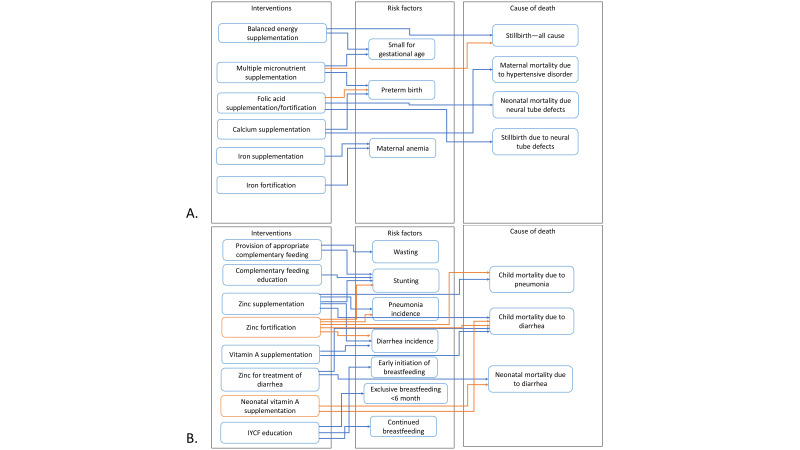
**Panel A:** Intervention-outcome pairs for women of reproductive age or pregnant women. **Panel B:** Intervention-outcome pair for infant or children 0-59 months. Blue boxes and arrows are existing interventions and links. Orange boxes and arrows are new interventions and links. IYCF-Infant and young child feeding

When we began the review, 12 nutrition interventions were included in LiST (**Figure 1**, Panel A and Panel B). We identified potential new interventions for inclusion and compiled and assessed available systematic reviews for each of the existing and potential interventions. This evidence was examined by four external advisors with extensive nutrition expertise, hereafter referred to as the External Advisory Group (EAG), to decide whether new information warranted a change to LiST parameters. In some cases, more in-depth analyses of trial data included in the meta-analyses were needed for the group to decide what to include in LiST.

The review considered a variety of interventions, including delivering food or nutrient through supplementation or fortification, social/behaviour change interventions that might influence food-related or child feeding practices, and agricultural or social protection interventions that might affect food accessibility or affordability at the population level or in a population sub-group. Treatment for acute malnutrition was not reviewed because there were no new data. Potential new interventions were suggested by EAG members, based either on their expertise or the recommendation of the interventions by the World Health Organization (WHO).

A quality score for each intervention-outcome (I-O) pair was developed using modified GRADE methods to assess the available systematic reviews [9]. Although most systematic reviews already provided quality grading for each outcome, LiST has additional quality considerations. A LiST-specific quality checklist (presented in section three of **Online Supplementary Document**) was developed to facilitate consistent decisions during the I-O pair reviews. The types of reviews that were considered included systematic reviews limited to RCTs only, reviews that were a mix of RCTs and non-RCTs, and reviews which contained non-RCTs only. Reviews based on RCTs only started off with a high-quality score, the mix of RCTs and non-RCTs started as moderate quality, and non-RCTs only started off with low-quality grading. The quality of the evidence was further assessed and downgraded by one or two levels for serious, or very serious limitations based on five criteria: indirectness, risk of bias, inconsistency, imprecision, and publication bias.

Indirectness is the degree to which the systematic review included studies that are not of direct relevance to LiST, ie, not conducted in settings or among populations with a high likelihood of benefit; it was the most important criterion applied in the review. We assessed directness according to the population groups studied and context. Appropriate population groups varied by intervention and included women of reproductive age (15-49 years), pregnant women, infants (<6 months), and children (6-59 months). The inclusion of studies carried out in LMIC and studies with a clear definition of intervention and comparison groups was also important for directly estimating intervention effects on cause-specific mortality, birth outcomes, and child anthropometry,

For risk of bias, we relied on the judgement of the systematic reviews because we did not evaluate all the individual studies included for each outcome. Inconsistency was based on variation in the effect estimates across studies according to the *I^2^* value. Imprecision was graded based on the number of studies included in the review, the total number of outcome events that occurred, and the width of the confidence interval. Publication bias was graded based on the funnel plot or statistical tests when available. When the systematic review did not provide funnel plots, publication bias was assessed according to the number of included studies with small sample sizes and significant effect sizes. When the quality was not downgraded previously, it could be upgraded if the effects were large, or a dose-dependent response was documented.

More in-depth analyses were conducted when necessary. We conducted a re-analysis of studies that provided supplemental complementary food because two of the available systematic reviews [10,11] included “any type” of complementary food, while another review [12] included only a small-quantity lipid-based nutrient supplement (SQ-LNS). All three reviews compared one type of complementary food to a control group that received no supplement. We performed a new meta-analysis including a subset of individual studies included in the 3 meta-analyses (reasons for excluding certain studies were explained in the **Online Supplementary Document**) and carried out separate subgroup analyses of three types of complementary food: local food without any fortification; prepared food that is industrially processed and fortified but is not an LNS; and SQ-LNS.

The EAG reviewed all findings and further analyses independently and discussed their conclusions in four 90-minute sessions. When one I-O pair had more than one systematic review, we chose the one with a higher quality score (even if not the most recent). When the quality scores were the same, we chose the one with higher directness. For the potential new interventions, judgment for inclusion was based on the quality of evidence, any reported potential adverse effects, and whether the relevant outcome was linked to mortality. Any disagreements about how the evidence should be interpreted were resolved during the four sessions.

For the existing I-Opairs, the EAG proposed effect sizes and affected fractions. For the new interventions, the group first decided if there was sufficient evidence for an effect of the intervention on a relevant outcome, and if so, recommended the efficacy and affected fractions.

Once a decision was made on the appropriate evidence for the intervention-outcome pair, we converted the reported effect size to the efficacy (1-relative risk), or odds ratio used in LiST. Odds ratios are applied to outcomes with distributions, including the prevalence of stunting, wasting, and breastfeeding practices. For example, if an intervention reduces wasting, the impact is expressed as reduced odds of moving to a more severe level of wasting. A detailed description of these conversion calculations is available in section two of the **Online Supplementary Document**. We also checked data sources to update the affected fractions, as needed. We searched online and consulted with the EAG on sources of updated data on affected fractions for different intervention-outcome pairs, using indicators such as food insecurity, maternal anaemia, and zinc deficiency. Our primary objective was to improve the way that LiST applies intervention efficacy estimates, restricting estimations to populations with similar risk profiles and potential to benefit, based on the evidence from the trials.

## RESULTS

Overall, the group reviewed 53 I-O pairs, including 25 I-O pairs already modelled in LiST and 28 new I-O pairs. Both efficacy and affected fractions were updated for seven pairs, efficacy only was updated for three pairs, and affected fractions only were updated for three pairs. Nine new I-O pairs were added. The latter included two new pairs for maternal nutrition interventions, five new pairs for a new intervention-zinc fortification, and two new pairs for a new intervention-neonatal vitamin A supplementation.

**Table 1** summarizes the 34 I-O pairs now included in LiST, their updated efficacy and affected fractions, and the quality of evidence supporting these decisions. Tables S1 and S2 in the **Online Supplementary Document** summarize the systematic review quality ratings for I-O pairs for women of reproductive age or pregnant women and for infants and children 0-59 months, respectively.

**Table 1:** Summary of the 34 nutrition-related intervention-outcome pairs in LiST

Intervention	Outcome	Efficacy (95% CI)*	Reference	Affected fraction	Quality
**Existing intervention-outcome pairs for women of reproductive age or pregnant women**					
Iron fortification	Maternal anaemia†	0.34 (0.24, 0.44)	Keats et al., 2019 [13]	Percent of WRA who are iron deficient‡	Very low
Folic acid fortification/supplementation	Stillbirth due to neural tube defects	0.41 (0.32, 0.48)	Imdad et al., 2011 [14]	Percent of WRA who are folate insufficient‡	Very low
Folic acid fortification/supplementation	Neonatal mortality due to neural tube defects	0.41 (0.30, 0.51)	Keats et al., 2019 [13]	Percent of WRA who are folate insufficient‡	Very low
Balanced energy protein supplementation	Small for gestational age birth	0.29 (0.06, 0.46)§	Lassi et al., 2020 [15]	Percent of PW who are food insecure‡	Low
Multiple micronutrient supplementation	Small for gestational age birth	0.07 (0.02, 0.12)§	Oh et al., 2020 [16]	All PW‡	Moderate
Calcium supplementation	Preterm birth	0.19 (-0.02, 0.36)§	Hofmeyr et al., 2018 [17]	Percent of PW who are calcium deficient‡	High
Multiple micronutrient supplementation	Preterm birth	0.05 (-0.01, 0.10)§	Keats et al., 2019 [18]	All PW‡	Moderate
Iron supplementation	Maternal anaemia†	0.70 (0.54, 0.81)§	Pena-Rosa et al., 2015 [19]	Percent of PW who are iron deficient‡	Moderate
Iron fortification	Maternal anaemia†	0.27 (0.16, 0.36)§	Keats et al., 2019 [13]	Percent of PW who are iron deficient‡	Very low
Balanced energy protein supplementation	Stillbirth	0.61 (0.20, 0.81)§	Lassi et al., 2020 [15]	Percent of PW who are food insecure‡	Moderate
Calcium supplementation	Maternal mortality due to hypertensive disorder	0.20 (0.02, 0.34)	Hofmeyr et al., 2018 [17]	Percent of PW who are calcium deficient‡	Low
**Existing intervention-outcome pairs for infant and children 0-59 mo**					
Infant and young child feeding education	Early initiation of breastfeeding	OR = 1.82 (1.32, 2.50) when delivered in health system; OR = 3.38 (1.97, 5.90) when delivered in home/community; OR = 4.96 (2.88, 8.54) when delivered in combined settings	Sinha et al., 2017 [20]	All infants <1 mo	Very low
Infant and young child feeding education	Exclusive breastfeeding <1 mo	OR = 2.03 (1.33, 3.10) when delivered in health system; OR = 2.17 (1.84, 2.56) when delivered in home/community; OR = 2.33 (0.85, 6.45) when delivered in combined settings	Sinha et al., 2017 [20]	All infants <1 mo	Low
Infant and young child feeding education	Exclusive breastfeeding 1-6 mo	OR = 3.07 (2.09, 4.52) when delivered in health system; OR = 2.48 (1.99, 3.09) when delivered in home/community; OR = 6.8 (3.75, 12.33) when delivered in combined settings	Sinha et al., 2017 [20]	All infants 1-6 mo	Low
Infant and young child feeding education	Continued breastfeeding 6-23 mo	OR = 1.42 (0.88, 2.28) when delivered in health system; no estimates for home/community; OR = 1.42 when delivered in combined settings	Sinha et al., 2017 [20]	All infants and young children 6- 23 mo	Very low
Complementary feeding education only	Stunting	OR = 1.3 (1.1, 1.5) for no education	Panjwani et al., 2016 [10]	Percent of children 6-23 mo who are food secure‖	High
Provision of appropriate fortified complementary food¶	Stunting	OR = 1.66 (1.48, 1.86) with the intervention; OR = 1.92 (1.64, 2.28) without the intervention§	Dewey et al., 2021 [12]	Percent of children 6-23 mo who are food insecure‖	High
Provision of appropriate fortified complementary food¶	Wasting	OR = 1.5 with the intervention; OR = 1.64 without the intervention§	Dewey et al., 2021 [12]	Percent of children 6-23 mo who are food insecure‖	High
Zinc supplementation	Stunting	OR = 1.11 (1.04, 1.20) for no zinc supplementation	Bhutta et al., 2013 [4]	Percent of children 12-59 mo who are zinc deficient	High
Zinc supplementation	Diarrhoea incidence	0.65 (0.48, 0.77)	Black et al., 2013 [21]	Percent of children 12-59 mo who are zinc deficient	Moderate
Vitamin A supplementation	Diarrhoea incidence	0.38 (0.13, 0.56)	Black et al., 2013 [21]	Percent of children 6-59 mo who are vitamin A deficient	Low
Zinc supplementation	Pneumonia incidence	0.52 (0.28, 0.68)	Black et al., 2013 [21]	Percent of children 12-59 mo who are zinc deficient	High
Zinc for treatment of diarrhoea	Neonatal mortality due to diarrhoea	0.23 (0.15, 0.31)	Walker et al., 2010 [22]	Infants <1 mo with diarrhoea	Moderate
Zinc for treatment of diarrhoea	Child mortality due to diarrhoea	0.23 (0.15, 0.31)	Walker et al., 2010 [22]	Children 6-59 mo with diarrhoea	Moderate
Zinc supplementation	Child mortality due to diarrhoea	0.50 (-0.25, 0.73)	Black et al., 2013 [21]	Percent of children 12-59 mo who are zinc deficient	Moderate
Zinc supplementation	Child mortality due to pneumonia	0.49 (-0.28, 0.80)	Black et al., 2013 [21]	Percent of children 12-59 mo who are zinc deficient	Moderate
Vitamin A supplementation	Child mortality due to diarrhoea	0.53 (0.35, 0.66)	Black et al., 2013 [21]	Percent of children 6- 59 mo who are vitamin A deficient	High
**New intervention-outcome pair for women of reproductive age or pregnant women**					
Folic acid fortification/supplementation	Preterm birth	0.12 (0.09, 0.15)	Li et al., 2019 [23]	Percent of WRA who are folate insufficient	Very low
Multiple micronutrient supplementation	Stillbirth	0.09 (0.02, 0.15)	Oh et al., 2020 [16]	All pregnant women	High
**New intervention-outcome pairs received by infants and children 0-59 mo**					
Neonatal Vitamin A supplementation	Neonatal mortality due to diarrhea	0.13 (0.06, 0.20)	Neonatal Vitamin A supplementation evidence group, 2019 [24]	Percent of PW who are vitamin A deficient	Moderate
Neonatal Vitamin A supplementation	Child mortality due to diarrhea	0.13 (0.06, 0.20)	Neonatal Vitamin A supplementation evidence group, 2019 [24]	Percent of PW who are vitamin A deficient	Moderate
Zinc fortification	Diarrhea incidence	0.65 (0.48, 0.77)	Black et al., 2013 [21]	Percent of children 12-59 mo who are zinc deficient	Low
Zinc fortification	Pneumonia incidence	0.52 (0.28, 0.68)	Black et al., 2013 [21]	Percent of children 12-59 mo who are zinc deficient	Moderate
Zinc fortification	Stunting	OR = 1.11 (1.04, 1.20) for no zinc fortification	Bhutta et al., 2013 [4]	Percent of children 12-59 mo who are zinc deficient	Moderate
Zinc fortification	Child mortality due to diarrhea	0.50 (-0.25, 0.73)	Black et al., 2013 [21]	Percent of children 12-59 mo who are zinc deficient	Low
Zinc fortification	Child mortality due to pneumonia	0.49 (-0.28, 0.20)	Black et al., 2013 [21]	Percent of children 12-59 mo who are zinc deficient	Low

CI – confidence interval, PW – pregnant women, WRA – non-pregnant women of reproductive age, 15-49 years old, IYCF – infant and young child feeding, OR – odds ratio, mo – months

*If not specified, efficacy is calculated as 1-relative risk; OR is also used in the model to estimate impact of an intervention.

†Outcome changed from iron-deficiency anaemia to all anaemia.

‡Updated affected fraction.

§Updated efficacy.

‖Updated data source for the affected fraction.

¶The estimates of efficacy from the meta-analysis on SQ-LNS trials was used for the intervention.

**Table 2** summarizes the 19 I-O pairs that were reviewed, but not included in LiST at this time. Of those, seven were excluded due to a lack of significant impact on the outcome of interest. Three I-O pairs for interventions addressing child anaemia were excluded because of the lack of evidence of its association with mortality [13,32-34]. Other reasons for excluding I-O pairs included the lack of systematic reviews available on the association of interest, pooled estimates derived from very small study populations (n <400) [38], an inability to model the impact due to the way data were presented [29]; the lack of clarity in the definition of the intervention (nutrition-sensitive agriculture which may include more than one intervention – eg, agriculture inputs and BCC) [31], the absence of country-level data on the outcome of interest (eg, child mortality due to neural tube defects), or the review’s failure to provide estimated impacts of the intervention (eg, the direct impact of SQ-LNS on cause-specific child mortality other than the indirect effect via stunting and wasting). One I-O pair was also excluded because the impact of calcium supplementation on pre-eclampsia might be accounted for in other existing links (calcium and preterm birth, and calcium and maternal mortality due to hypertensive disorder) [17].

**Table 2:** Summary of the 19 intervention-outcome pairs excluded and reason for exclusion

Intervention	Outcome	Reference	Quality	Reason for exclusion
**Intervention-outcome pairs for women of reproductive age or pregnant women**				
Periconceptual folic acid fortification	Child mortality due to neural tube defects	Blencowe et al., 2018 [25]	Very low	Lack of information on proportion of child mortality due to neural tube defects
Zinc fortification	Preterm birth	Carducci et al., 2021 [26]	Low	Evidence from supplementation trials showed insignificant impact
Omega-3 fatty acid supplementation	Preterm birth	Middleton, 2018 [27]	Moderate	Evidence showed insignificant impact and potential adverse impact
Vitamin D supplementation	Preterm birth	Palacios et al., 2018 [28]	Low	Evidence showed insignificant impact and potential adverse impact
Stop smoking education	Preterm birth	Chamberlain et al., 2017 [29]	Moderate	Inability to model due to the way data were presented. Evidence showed insignificant impact on preterm birth and significant impact on low birth weight, but the impact on SGA was not reported
Deworming	Maternal anaemia	Salam et al., 2021 [30]	Low	Evidence showed insignificant impact
Calcium supplementation	Pre-eclampsia	Hofmeyr et al., 2018 [17]	Low	The impact was probably accounted in the link between calcium and preterm birth
Thiamine supplementation	Neonatal mortality	NA	NA	No available systematic review
**Intervention-outcome pairs for infants and children 0-59 mo**				
Nutrition sensitive agriculture intervention	Appropriate complementary feeding	Margolies et al., 2022 [31]	Very low	No standard definition of the intervention
Provision of SQ-LNS	Child anaemia	Wessells et al., 2021 [32]	Moderate	Lack of evidence on link between child anemia and mortality
Multiple micronutrient powder	Child anaemia	Suchdev et al., 2020 [33]; De-regil et al., 2017 [34]	Moderate	Lack of evidence on link between child anemia and mortality
Iron fortification	Child anaemia	Keats et al., 2019 [13]	Very low	Lack of evidence on link between child anemia and mortality
Deworming	Wasting	Thayer et al., 2017 [35]	Very low	Evidence showed insignificant impact
Prophylactic antibiotics	Stunting	NA	NA	No available systematic review
Vitamin D supplementation	Pneumonia incidence	Martineau et al., 2017 [36]	Low	Evidence showed insignificant impact
Vitamin D supplementation	Stunting	Huey et al., 2020 [37]	Low	Evidence showed insignificant impact
Neonatal zinc supplementation	Neonatal mortality due to sepsis	Irfan et al., 2022 [38]	Very low	Very small study population (n <300)
Zinc for treatment of sepsis	Neonatal mortality due to sepsis	Irfan et al., 2022 [38]	Very Low	Very small study population (n <400)
Provision of SQ-LNS	Child mortality	Stewart et al., 2020 [39]	Moderate	The review examined MQ-LNS and SQ-LNS together and estimated direct impact of SQ-LNS on cause-specific mortality was not available.

NA – not applicable, MQ-LNS – median-quantity lipid-based nutrient, SQ-LNS – small-quantity lipid-based nutrient supplementation

In the past, the benefits of vitamin A and zinc supplementation were applied only to affected fractions, ie, children who are vitamin A or zinc deficient. In this review, the benefit of all the other preventive interventions providing a macro- or micro-nutrient supplement, like calcium, iron, or folic acid for pregnant women, were likewise applied just to the percentage of the population deemed to be deficient/insufficient in the specific macro/micronutrient. The rationale for this decision is that the efficacy of the intervention was also determined based on trials conducted among populations at high risk of deficiency or was adjusted to attribute the impact to the deficient fraction of the population.

**Figure 2:**
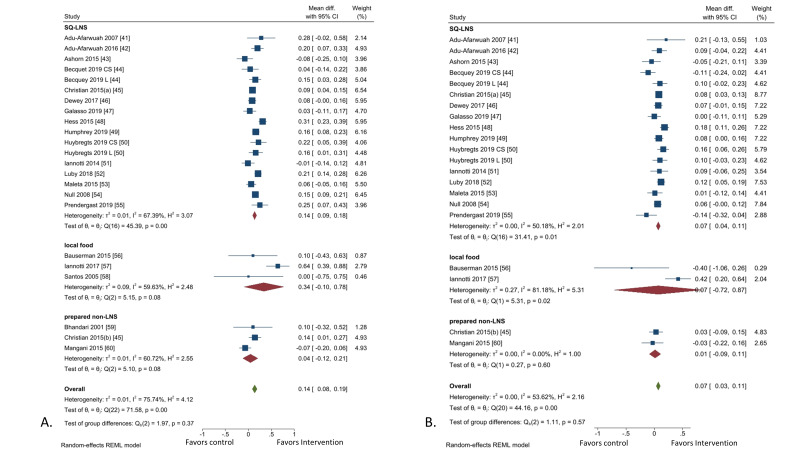
Provision of complementary food vs no supplemental complementary food. **Panel A:** mean difference in height-for-age z-score. **Panel B:** mean difference in weight for height z-score. Iannotti 2017 [56] provided egg.

Our new meta-analysis on the effects of supplemental complementary foods [40-59] showed that, of the three different types of complementary food, only SQ-LNS has a significant benefit on height-for-age z-score (HAZ) and weight-for-height z-score (WHZ) (**Figure 2**, Panels A and B). Given that 1) national programs for the provision of SQ-LNS are not yet widely available in LMICs, and 2) there are only six studies on other complementary foods that are not LNS, we decided not to restrict the intervention to the provision of SQ-LNS only, but rather to call the intervention “Provision of appropriate fortified complementary food”. The estimates of efficacy from the meta-analysis on SQ-LNS trials were used for this intervention (**Table 1**). A recent review that examined the impact of SQ-LNS on different levels of wasting and stunting [60] found that SQ-LNS reduces severe wasting (31%) and severe stunting (17%) more than it affects moderate wasting (12%) and moderate stunting (14%). Since severe wasting and stunting result in greater mortality impact, the EAG also recommended separate assessments of the impact of SQ-LNS depending on the levels of wasting and stunting. We are currently working with programmers to incorporate these different categories of wasting and stunting in the model. Meanwhile, we used the combined categories of overall wasting and stunting in the model (**Table 1**).

Food insecurity as a measure for limited access to sufficient, safe, and nutritious food characterizes the subset of the population that can likely benefit from nutrition interventions that provide supplemental food. Previously, the food insecurity indicator used in LiST was the percentage of the population living below $ 1.90 a day, which is an indicator of poverty. With the Food Insecurity Experience Scale (FIES) indicator now available for 77 countries (from 2014-2018, reported in three-year averages) [61] and included in the regular Gallup Polls data collection [62], we decided to use FIES rather than an income-related poverty indicator in LiST as this metric has been shown through validation studies to accurately reflect different levels of severity of food insecurity in LMICs populations [63]. However, FIES data were missing for several countries, including India and Pakistan, which account for roughly 20% of the total LMICs population. To assess whether the percentage of the population living on<$1.90/d could be used for countries where FIES data were unavailable, we performed a correlation analysis in countries where both data were available. We found a strong correlation between FIES and the percentage of the population living on<$1.90/d (Pearson correlation coefficient = 0.82) and decided to use the percentage of the population living on<$1.90/d for countries when FIES data are unavailable.

We added two new I-O pairs for maternal nutrition interventions (**Figure 1**, Panel A). For non-pregnant women of reproductive age, a meta-analysis based on eight observational studies concluded that periconceptional folic acid supplementation reduced the risk of preterm birth [23]. There are few national programs for folic acid supplementation before pregnancy and more programs for folic acid fortification; hence we decided to model the impact of either periconceptional folic acid supplementation or fortification. For consistency, we named this intervention folic acid supplementation/fortification.

For pregnant women, we added a new I-O pair for multiple micronutrient supplementation (MMN) and stillbirth (**Table 1**). The evidence on MMN and stillbirth was reported in three systematic reviews. Two older reviews included a similar set of studies, and the impact was significant in a fixed effects model but not in a random effects model [18,64]. The most recent review mistakenly included a large calcium trial [16], but provided a subgroup analysis of trials using MMN containing greater than four micronutrients, which included a similar set of studies as in the other two reviews and found a significant impact on stillbirths in a random effects model [16].

Two new interventions for children, zinc fortification and neonatal vitamin A supplementation were added to LiST. Although no direct evidence was available for zinc fortification and health outcomes, a recent meta-analysis found that consumption of zinc-fortified food increased plasma/serum zinc concentration, with a corresponding decrease in the prevalence of zinc deficiency [65]. We applied efficacy estimates from zinc supplementation trials to model the impact of zinc fortification on zinc-deficient children. As a result, five additional intervention-outcome pairs were added (**Figure 1**, Panel B).

Receipt of vitamin A within the first 2-3 days after birth is effective at reducing all-cause mortality in infants <6 months of age [24]. Based on the evidence, WHO provided a context-specific recommendation (settings with the prevalence of VAD deficiency ≥10% and infant mortality rate >50 per 1000 live births) for the intervention [66]. Most South Asian countries did not meet these criteria, including the countries where a benefit was demonstrated. Therefore, we decided not to adopt the WHO context criteria. Instead, we applied this intervention using the percentage of vitamin A-deficient pregnant women as our affected fraction. Since only cause-specific mortality is modelled in LiST and vitamin A was effective at reducing diarrhoea, the intervention was applied only to mortality due to diarrhoea for infants <6 months old. It was noted that vitamin A supplementation for children 6-59 months is a different intervention in the model and was applied to mortality in 6-59-month-olds due to diarrhoea (**Table 1**).

## DISCUSSION

The 34 nutrition intervention-outcome pairs in LiST include 14 interventions (six for women of reproductive age and pregnant women and eight for infants and children) and 16 nutrition, disease incidence and cause-specific mortality outcomes (**Figure 1**, Panel A and B). The present updates to the selected interventions, the magnitude of their impact, and affected fractions will enable LiST users to better estimate the potential health, nutrition, and survival benefits of investing in nutrition.

Much has been written about the relatively low cost and high return on investment that can be achieved by investing in proven nutrition interventions [4,67,68], but such investments have not reached their full potential. The 2021 Tokyo Nutrition for Growth Summit garnered $27 billion in new, multi-year pledges of support to address all forms of malnutrition, falling well short of the $10.8 billion needed annually to achieve the 2030 global maternal and child undernutrition SDG targets [69]. The global nutrition investment gap is likely to widen even further with the combined challenges posed by COVID-19, the conflict in Ukraine, and climate change [70-73], underscoring the need for governments to make better use of available resources while aiming to increase overall levels of investments in nutrition.

LiST can help decision-makers prioritize the most impactful interventions in their population at specific time points. A strength of the tool is the routine updating of interventions, values for efficacy, and affected fraction based on evolving scientific evidence. The tool allows for the calculation of benefits of interventions when only some population sub-groups can actually benefit from them. For example, the benefits of neonatal vitamin A supplementation can be estimated for the subset of infants born to vitamin A deficient mothers, even in populations where the prevalence of vitamin A deficiency is less than the threshold proposed by the current WHO recommendation. Because LiST uses cause-specific mortality, the impact of the interventions can account for the varying causes of death over time and the difference from the patterns that occurred at the time of the original trials. For example, diarrhoea and measles are now responsible for a much smaller proportion of child deaths compared to 20 years ago [74], so the mortality impact of interventions that reduce the risk of these illnesses is expected to be less than at the time of the trials – LiST takes these time-related changes into account.

LiST also has some limitations. Sufficient evidence is not always available for potential interventions with likely beneficial impacts, so certain assumptions and proxies are sometimes used in the model. For example, results from nutrient supplementation trials may be extrapolated to fortification interventions, as was done in the case of zinc fortification. We acknowledge that more research is needed to better understand if zinc fortification performs the same as supplementation, but recent observations confirming the positive impact of zinc fortification on serum zinc concentration allow for an assumption that other benefits observed with zinc supplementation will likewise accrue with zinc fortification interventions. The tool also relies on average estimates of efficacy observed in different trials, whereas benefits may vary across risk groups in a population. Nevertheless, LiST users have the flexibility to change estimates of efficacy and affected fraction based on their knowledge about a country or certain circumstances.

One limitation of this study is the lack of resources to conduct new systematic reviews. Relying on existing systematic reviews means that we had to compromise for certain intervention-outcome pairs, as described above for different types of complementary foods. These estimates will be updated as more evidence becomes available. A recent systematic review reported no difference in growth outcomes among children given fortified vs non-fortified complementary foods [75], but none of the included studies had a control arm receiving no supplemental complementary food.

In LiST, we use efficacy or odds ratio to estimate the impact of an intervention. However, the “efficacy” estimates used in LiST are not always drawn from true efficacy trials where everyone who should receive the intervention actually receives it. Sometimes our estimates are effectiveness studies in real-world contexts without a guarantee that everyone receives the intervention as planned. In some instances, where trial data were lacking, we used observational studies to estimate the impact. Using data from a mix of efficacy and effectiveness trials and treating them as efficacy is a limitation of the model. We accept this limitation because gold standard data are not always available in LMICs due to research resource constraints and ethical concerns (in some cases) about withholding interventions from a control group.

In this update, we encountered several challenges. One is how to best match the efficacy and the affected fraction. For example, in the most recent Cochrane review on calcium supplementation for pregnant women, the impact on preterm birth is statistically insignificant for trials in populations with low calcium intake, but the pooled impact is greater and significant for trials in populations with adequate or low calcium intake [17]. A previous systematic review [76] found a significant impact on the low calcium intake population. We believe that the evidence is sufficient to prove the effect, but we do not want to overestimate the impact. Therefore, we decide to keep the affected fraction as a calcium deficient population (also consistent with the WHO recommendation) and use the efficacy based on low calcium intake trials from the most recent Cochrane review.

Another challenge we faced for some interventions is how to identify the appropriate affected fraction and the data source for this information. For balanced energy protein supplementation (BEP) in pregnant women, for example, the study populations do not have clear and consistent characteristics that make them more responsive to the intervention. Some studies included only women with low body-mass index, whereas others characterized the study population as having a suboptimal nutritional status defined by low energy intake or nutritional deficiency. We decide that food insecurity best describes the population that can benefit from BEP, so this is the information used to determine the affected fraction for this intervention.

The conceptual basis for defining the affected fraction for micronutrient supplementation for children is more straightforward, as it can be based on the prevalence of a particular deficiency. However, it can be challenging to determine the prevalence of deficiency when there is sparse information on biomarkers of micronutrient status, as is the case with zinc. The recommended biomarkers for assessing zinc status are plasma or serum zinc concentration [77], but national-level data on plasma/serum zinc are not widely available in LMICs [78]. In lieu of this information, we applied data on the adequacy of zinc in the national food supply based on national food balance sheet data [58]. Affected fractions for all other nutrient deficiencies (except vitamin A deficiency among pregnant women) are based on rough proxies of inadequate intake, because true prevalence based on biomarker data are also not available [79].

Although we systematically looked for newer reviews (published after the last LiST update) to revise our effect sizes for the different interventions and outcomes, there are instances where we choose not to update the previous estimates because of concerns with the inclusion or exclusion criteria used in some of the new reviews. For example, two large recent systematic reviews on vitamin and mineral supplementation – one for pregnant women and one for children under five years – excluded studies published before 1995 [16,80]. This criterion excluded several older trials for calcium, iron, and vitamin A, not on the basis of poor quality, but on the basis of date of publication. We decide that excluding older studies is inappropriate because 1) vitamin and mineral supplementation impacts various health outcomes through biological pathways that are not deemed to be time-dependent and 2) the time varying prevalence of nutritional risk groups is already accounted for by the affected fraction in the model and secular changes in the distribution of causes of neonatal and child deaths included in LiST.

We found evidence for new intervention-outcome pairs. For child anaemia, there is sufficient evidence to support intervention effects of SQ-LNS, multiple micronutrient powder, and iron fortification (**Table 2** and **Online Supplementary Document**), but we decided not to include the intervention for now because LiST is a mortality focused model, meaning all the risk factors are linked to mortality. Currently, no evidence for child anaemia and risk of mortality exists. There is solid evidence for calcium supplementation and pre-eclampsia, but we cannot disentangle the linkages between calcium and preterm [17] or the association between pre-eclampsia and preterm birth [81]. We therefore decided not to include preeclampsia in LiST until we have more knowledge on how to handle the complicated associations between calcium, preeclampsia, and preterm birth. We recognize that pre-eclampsia is also a risk factor for maternal mortality, but this is beyond the scope of this study.

## CONCLUSIONS

LiST incorporates new scientific evidence as it becomes available, which is crucial for modelling tool to remain useful. In the present updates, we reviewed 53 intervention-outcome pairs to update LiST and the tool now has 34 nutrition intervention-outcome pairs (an increase from 25 before this review). The new set includes nine new intervention-outcome pairs, 13 existing links with updated efficacy and/or affected fractions, and 12 existing links with no changes to estimates of efficacy or affected fraction. Assessing quality of the available systematic reviews is an important step in the regular updates, especially when systematic reviews yield inconsistent estimates of intervention efficacy. It is important for systematic reviews to provide clear inclusion and exclusion criteria to help us to interpret the evidence and to identify the appropriate estimates of efficacy and population-affected fraction to use in LiST.

## Additional material: 


Online Supplementary Document
