# Supplementary material for: Maternal and child nutrition in the Lives Saved Tool: Results of a recent update
Source: J Glob Health. 2022 Dec 30;12:08005. doi: 10.7189/jogh.12.08005 (PMC9801341; doi:10.7189/jogh.12.08005)
Supplement: Online Supplementary Document [file jogh-12-08005-s001.pdf]

ONLINE SUPPLEMENTARY DOCUMENT

Title: Maternal and Child Nutrition in the Lives Saved Tool: Results of a recent update

Authors: Hannah Tong, Ellen Piwoz, Marie T. Ruel, Kenneth H. Brown, Robert E. Black, Neff Walker

Contents

Section One: Quality assignment and application in LiST 3

Table S1. Quality assignment and application in LiST for intervention-outcome pairs for women of reproductive age or pregnant women 3

Table S2. Quality assignment and application in LiST for intervention-outcome pairs for infant and children 0-59 months 7

Section Two: Description of the meta-analyses reviewed 16

Overview 16

Existing Interventions received by women of reproductive age (WRA) or pregnant women (PW) 16

Balanced energy protein supplementation (BEP)—stillbirth; SGA birth: 16

Calcium supplementation—preterm birth; maternal mortality due to hypertensive disorder; pre-eclampsia: 17

Iron with or without folic acid supplementation or multiple micronutrient supplementation during pregnancy and anemia—maternal anemia: 18

Multiple micronutrient supplementation during pregnancy (MMN)—preterm birth; SGA birth; stillbirth: 19

Iron fortification—maternal anemia: 20

Periconceptual folic acid supplementation/fortification—stillbirth due to neural tube defects; neonatal mortality due to neural tube defects; preterm birth; child mortality due to neural tube defects: 21

Potential new interventions for women of reproductive age or pregnant women 22

Vitamin D supplementation for pregnant women—preterm births; pre-eclampsia: 22

Stop smoking education for pregnant women who smoke—preterm birth: 23

Deworming for pregnant women—maternal anemia: 23

Maternal thiamine supplementation—neonatal mortality: 23

Zinc fortification—preterm birth: 23

Omega-3 fatty acid supplementation—preterm births: 24

Existing Interventions for infants and children 0-59 months 24

Infant and young child feeding (IYCF) education—early initiation of breastfeeding; exclusive breastfeeding; continued breastfeeding 24

Zinc for treatment of diarrhea—neonatal mortality due to diarrhea; child mortality due to diarrhea: 25

Complementary feeding education only—stunting: 26
Provision of appropriate fortified complementary foods—stunting; wasting; child mortality; child anemia: 26
Vitamin A supplementation-diarrhea incidence; child mortality due to diarrhea: 28
Zinc supplementation—diarrhea incidence; pneumonia incidence; child mortality due to diarrhea; child mortality due to pneumonia; stunting: 29
Potential new interventions for infants and children 0-59 months 30
Deworming for children 1-59 months—wasting: 30
Vitamin D for children 1-59 months—pneumonia incidence; stunting: 30
Prophylactic antibiotics for children 1-59 months—stunting: 31
Nutrition sensitive agriculture intervention—appropriate complementary feeding: 31
Zinc fortification—diarrhea incidence; pneumonia incidence; child mortality due to diarrhea; child mortality due to pneumonia; stunting: 31
Iron fortification—child anemia: 31
Multiple micronutrient powder for children—child anemia: 32
Neonatal vitamin A supplementation—neonatal mortality due to diarrhea; child mortality due to diarrhea: 32
Neonatal zinc supplementation—neonatal mortality due to sepsis: 33
Section Three: LiST-specific quality checklist 35
Section Four: Meta-analysis for provision of complementary food using loose criteria 37
Reference 38

Section One: Quality assignment and application in LiST

Table S1. Quality assignment and application in LiST for intervention-outcome pairs for women of reproductive age or pregnant women

Intervention	Outcome	Reference	Relative risk (95% CI); number of studies	Quality	Comments	Application in LiST (efficacy; affected fraction)
Existing Intervention-outcome pairs						
Balanced energy protein supplementation	Small for gestational age birth	Ota et al 2015	0.79 (0.69, 0.90); 7 studies	Low	Starting High; -1 for risk of bias; -1 for not all LMICs and allowed nonstandard of care control	NA
		Lassi et al 2020	0.71 (0.54, 0.94); 3 studies	Low	Starting Moderate; -1 for risk of bias	0.29; % of PW who are food insecure
Multiple micronutrient supplementation	Small for gestational age birth	Smith et al 2017	0.94 (0.90, 0.98); 16 studies	High	NA	NA
		Keats et al 2019	0.92 (0.88, 0.97); 17 studies	Moderate	Starting High; -1 for publication bias	NA
		Oh et al 2020	0.93 (0.88, 0.98); 19 studies	Moderate	Starting High; -1 for publication bias	0.07; all pregnant women
Multiple micronutrient supplementation	Preterm	Smith et al 2017	0.93 (0.87, 0.98); 16 studies	High	NA	NA
		Keats et al 2019	0.95 (0.9, 1.0); 18 studies	Moderate	Starting High; -1 for publication bias	0.05; all pregnant women
		Oh et al 2020	0.96 (0.91, 1.01); 29 studies	Moderate	Starting High; -1 for inclusion of inappropriate interventions	NA
Calcium supplementation	Preterm	Imdad et al 2011	0.88 (0.78, 0.99); 5 studies	High	NA	NA
		Hofmeyr et al 2018	0.81 (0.64, 1.02); 7 studies	High	NA	0.19; % PW who are calcium deficient

		Oh et al 2020	0.84 (0.65, 1.08); 3 studies	Moderate	Starting High; -1 for excluding studies before 1995	NA
Iron supplementation	Maternal anemia	Pena-Rosa et al 2015	0.3 (0.19, 0.46); 14 studies	Moderate	Starting High; -1 for risk of bias; -1 for inconsistency; +1 for large RR and dose-dependent gradient	0.7; % of PW who are iron deficient; same efficacy for MMN, but user can only choose one of the two interventions
		Oh et al 2020	0.53 (0.43, 0.65); 6 studies	Very low	Starting High; -1 for serious inconsistency; -1 for excluding studies before 1995	NA
Iron fortification	Maternal anemia	Keats et al 2019	0.66 (0.56, 0.76); 9 studies	Very low*	Starting Moderate; -2 for very serious inconsistency; -1 for wide range of interventions included	0.34; % of WRA who are iron deficient
Iron fortification	Maternal anemia	Keats et al 2019	0.73 (0.64, 0.84); 3 studies	Very low*	Starting Moderate; -2 for very serious inconsistency; -1 for wide range of interventions included	0.27; % of pregnant women who are iron deficient
Folic acid supplementation/fortification	Stillbirth due to neural tube defects	Imdad et al 2011	0.59 (0.52, 0.68); 11 studies	Very low*	Staring Low; -1 for serious inconsistency; -2 for all HIC evidence and outcome examined is neural tube defects incidence	0.41; % PW who are folate insufficient
Balanced energy protein	Stillbirth	Ota et al 2015	0.6 (0.39, 0.94); 5 studies	Low	Starting High; -1 for risk of bias; -1 for not all LMICs and	NA

supplementation					allowed nonstandard of care control	
		Lassi et al 2020	0.39 (0.19, 0.8); 3 studies	Moderate	Starting High; -1 for risk of bias	0.61; % of PW who are food insecure
Folic acid supplementation/fortification	Neonatal mortality due to neural tube defects	Keats et al 2019	OR=0.59 (0.49, 0.70); 8 studies	Very low	Starting Moderate; -1 for serious inconsistency; -1 for wide variation in definition of the intervention	0.41; % PW who are folate insufficient
Calcium supplementation	Maternal mortality due to hypertensive disorder	Hofmeyr et al 2018	0.8 (0.66, 0.98); 4 studies	Low	Staring High; -2 for only 1 large study had events	0.2; % PW who are calcium deficient
New Intervention-outcome pairs						
Zinc fortification	Preterm birth	Ota et al 2015	0.87 (0.77-0.98); 14 studies	Low	Starting High; -1 for risk of bias; -1 because intervention is zinc supplementation	NA
		Carducci et al 2021	0.87 (0.74-1.03); 21 studies	Low	Starting High; -1 for risk of bias; -1 because intervention is zinc supplementation	Do not include because evidence from supplementation trials found insignificant impact
Folic acid supplementation/fortification	Preterm birth	Li et al 2019	OR=0.87 (0.84, 0.91); 8 studies	Very low*	Starting Low; -1 for lack of adjustment for confounding factor; -1 for lack of evidence from LMICs	0.12; % of PW who are folic insufficient
Omega-3 fatty acid supplementation	Preterm birth	Middleton et al 2018	0.90 (0.8, 1.01); 18 studies	Moderate	Starting High; -1 for majority HIC evidence	Do not include because 1) evidence of no impact 2) potential

						adverse impact on prolonged gestation and unclear risk on stillbirth
Vitamin D supplementa tion	Preterm birth	Palacios et al 2019	0.66 (0.34, 1.3); 7 studies	Low	Starting High; -1 for risk of bias; -1 for wide CI	Do not include because 1) evidence of insignificant impact 2) unclear adverse impact when also taking calcium
Stop smoking education	Preterm birth	Chamberl ain et al 2020	0.93 (0.77,1.11); 17 studies	Moderate	Starting High; -1 for all HIC evidence	Do not include because insignificant impact on outcome of interest
Deworming	Maternal anemia	Salam et al 2021	0.85 (0.72, 1.00); 5 studies	Low	Starting High; -1 for risk of bias; -1 for inconsistency Also see Thayer et al 2017, where deworming did not show consistent benefits for anemia in WRA	Do not include because insignificant impact
Calcium supplementa tion	Pre-eclampsi a	Hofmeyr et al 2018	0.36 (0.2, 0.65); 8 studies	Low	Starting High; -1 for serious inconsistency; -1 for publication bias	The impact was probably accounted in the link between Calcium and preterm birth
		Oh et al 2020	0.45 (0.19, 1.06); 4 studies	Very low	Starting High; -1 for serious inconsistency; -1 for excluding studies before 1995; -1 for publication bias	NA

Multiple micronutrient supplementation	Stillbirth	Smith et al 2017	0.97 (0.85, 1.11); 16 studies	High	NA	NA
		Keats et al 2019	0.95 (0.86, 1.04); 17 studies	High	NA	NA
		Oh et al 2020	0.91 (0.85, 0.98); 17 studies	High	NA	0.09; all pregnant women
Folic acid supplementation/fortification	Child mortality due to neural tube defects	Blencowe et al 2018	NA	NA	NA	Do not include because no data on proportion of child mortality due to neural tube defects

Very low*: the quality is downgraded to lower than very low
LiST-Lives Saved Tool, WRA-non-pregnant women of reproductive age 15-49 years, PW-Pregnant women, PTB-Preterm birth

Table S2. Quality assignment and application in LiST for intervention-outcome pairs for infant and children 0-59 months

Intervention	Outcome	Reference	Relative risk (95% CI); number of studies	Quality	Comments	Application in LiST (efficacy; affected fraction)
Existing intervention-outcome pairs						
Infant and young child feeding education	Early initiation of breastfeeding	Sinha et al 2017	Health system: OR=1.82 (1.32, 2.50); Home/community: OR=3.38 (1.97, 5.90); Combined: OR=4.96 (2.88-8.54); 22 studies	Very low	Starting Moderate; -2 for very serious inconsistency	Health system: OR=1.82; Home/community: OR=3.38; Combined: OR=4.96; all infants <1 months
		Lassi et al 2020	Facility-based: 1.18 (1.03, 1.36);	Very low	Starting High; -2 for very serious inconsistency; -1 for unclear	NA

			home/community-based: 1.17 (1.07, 1.28); 14 studies		description on different delivery setting of the intervention	
Infant and young child feeding education	Exclusive breastfeeding <1 month	Sinha et al 2017	Health system: OR=2.03 (1.33, 3.10); Home/community: OR=2.17 (1.84, 2.56); Combined: OR=2.33 (0.85-6.45); 19 studies	Low	Starting Moderate; -1 for serious inconsistency	Health system: OR=2.03; Home/community: OR=2.17; Combined: OR=2.33; all infants <1 months
		Lassi et al 2020	Facility-based: 4.30 (1.97, 6.21); home/community-based: 1.90 (1.76, 2.04); 6 studies	Very low*	Starting High; -2 for very serious risk of bias; -1 for inconsistency; -1 for the outcome is EBF<3 months and no clear definition of the outcome	NA
Infant and young child feeding education	Exclusive breastfeeding 1-6 months	Sinha et al 2017	Health system: OR=3.07 (2.09, 4.52); Home/community: OR=2.48 (1.99, 3.09); Combined: OR=6.8 (3.75-12.33); 44 studies	Low	Starting Moderate; -1 for inconsistency;	Health system: OR=3.07; Home/community: OR=2.48; Combined: OR=6.8; all infants 1-6 months
		Lassi et al 2020	Facility-based:	Very low*	Starting High, -2 for very serious	NA

			1.20 (1.15, 1.25); home/community-based: 1.90 (1.80, 2.00); 19 studies		risk of bias; -1 for inconsistency; -1 for lack of clear definition for different delivery setting of the intervention and the outcome	
Infant and young child feeding education	Continued breastfeeding 6-23 months	Sinha et al 2017	Health system: OR=1.42 (0.88, 2.28); Home/community: No estimates; Combined: OR=1.42; 7 studies	Very low	Starting Moderate; -1 for inconsistency; -1 for small number of studies included and lacking estimates/confidence interval and insignificant CI	Health system: OR=1.42 Home/community: no estimates; Combined: OR=1.42 all infants and young children 6-23 months
		Moore et al 2016	1.5 (1.18, 1.9); 7 studies	Very low	Starting High; -1 for risk of bias; -2 for excluding preterm/LBW infants and not all LMICs	NA
Zinc supplementation	Diarrhea incidence	Yakoob et al 2011	0.87 (0.81, 0.94); 15 studies	Moderate	Starting High; -1 for inconsistency	NA
		Black et al 2013	0.8 (0.73, 0.89); 13 studies	Moderate	Starting High; -1 for inconsistency RR attributed to zinc deficient fraction is 0.35 (0.23, 0.52)	0.65; % of children 12-59 months who are zinc deficient
		Tam et al 2020	0.89 (0.82, 0.97); 11 studies	Very low	Starting Moderate; -1 for inconsistency; -1 for excluding studies before 1995	NA
Zinc supplementation	Pneumonia incidence	Yakoob et al 2011	0.81 (0.73, 0.90); 6 studies	High		NA

		Black et al 2013	0.77 (0.65, 0.91); 3 studies	High	RR attributed to zinc deficient fraction is 0.48 (0.32, 0.72)	0.52; % of children 12-59 months who are zinc deficient
		Tam et al 2020	0.78 (0.49, 1.24); 6 studies	Low	Starting Moderate; -1 for inconsistency; -1 for wide CI	NA
Vitamin A supplementation	Diarrhea incidence	Black et al 2013	0.72 (0.72, 1.00); 12 studies	Low	Starting High; -1 for risk of bias; -1 for inconsistency RR attributed to vitamin A deficient fraction is 0.62 (0.44, 0.87)	0.38; % children 6-59 months who are vitamin A deficient
		Imdad et al 2017	0.85 (0.82, 0.87); 15 studies	Low	Starting High; -1 for risk of bias; -1 for inconsistency	NA
		Tam et al 2020	0.97 (0.83, 1.14); 7 studies	Very low*	Starting Moderate; -1 for risk of bias; -1 for inconsistency; -1 for excluding studies before 1995	NA
Complementary feeding education only	Stunting (HAZ)	Panjwani et al 2016	MD=0.22 (0.08, 0.37); 4 studies	High	NA	OR=1 for food secure children 6-23 month with education; OR=1.3 for food secure children 6-23 month without education
		Lassi et al 2020	MD=0.29 (0.04, 0.54); 4 studies	Moderate	Starting High; -1 for unclear classification of study population	NA
Provision of appropriate	Wasting (WHZ)	Panjwani et al 2016	MD=0.07 (0.03,	High	NA	NA

fortified complementary food			0.12); 4 studies			
		Lassi et al 2020	MD=0.01 (-0.01, 0.03); 10 studies	Moderate	Starting High; -1 for unclear classification of study population	NA
		Dewey et al 2021	MD=0.07 (0.03, 0.11); 14 studies	High	This review is SQ-LNS only	OR=1 for food secure children 6-23 months with or without education; OR=1.5 for food insecure children 6-23 months with complementary food; OR=1.64 for food insecure children 6-23 months without complementary food
Provision of appropriate fortified complementary food	Stunting (HAZ)	Panjwani et al 2017	MD=0.10 (0.03, 0.17); 7 studies	Moderate	Starting Moderate	NA
		Lassi et al 2020	MD=0.12 (0.05, 0.19); 10 studies	Moderate	Starting High; -1 for unclear classification of study population	NA
		Dewey et al 2021	MD=0.13 (0.09, 0.18); 14 studies	High	This review is SQ-LNS only	OR=1.66 for food insecure children 6-23 months with complementary food; OR=1.92 for food insecure children 6-23 month without complementary food

Zinc supplementation	Stunting	Bhutta et al 2013	OR=0.90 (0.83, 0.96); 27 studies	High	NA	OR=1.11 for no zinc supplementation; % of children 12-59 months who are zinc deficient
		Tam et al 2020	1.00 (0.89, 1.14); 6 studies	Very low	Starting moderate; -1 for including children<12; -1 for excluding studies before 1995	NA
Zinc for treatment of diarrhea	Neonatal mortality due to diarrhea	Walker & Black et al 2010	0.77 (0.69, 0.85); 2 studies	Moderate	Starting High; -1 for only in Asia and outcome is diarrhea hospitalization	0.23; neonates <1 month with diarrhea
		Lazzerini et al 2016	0.31 (0.09, 1.07); 8 studies	Low	Starting High; -1 for no cause-specific mortality; -1 for insufficient events	NA
Zinc for treatment of diarrhea	Child mortality due to diarrhea	Walker & Black et al 2010	0.77 (0.69, 0.85); 2 studies	Moderate	Starting High; -1 for only in Asia and outcome is diarrhea hospitalization	0.23; children 6-59 months with diarrhea
		Lazzerini et al 2016	0.31 (0.09, 1.07); 8 studies	Low	Starting High; -1 for no cause-specific mortality; -1 for insufficient events	NA
Zinc supplementation	Child mortality due to diarrhea	Yakoob et al 2011	0.83 (0.64, 1.05); 4 studies	Moderate	Starting High; -1 for wide CI	NA
		Black et al 2013	0.81 (0.63, 1.03); 3 studies	Moderate	Starting High; -1 for wide CI<br>RR attributed to zinc deficient fraction is 0.50 (0.27, 1.25)	0.50; % of children 12-59 months who are zinc deficient

Zinc supplementation	Child mortality due to pneumonia	Yakoob et al 2011	0.85 (0.65, 1.11); 4 studies	Moderate	Starting High; -1 for wide CI	NA
		Black et al 2013	0.87 (0.67-1.14); 3 studies	Moderate	Starting High; -1 for wide CI<br>RR attributed to zinc deficient fraction is 0.51 (0.20, 1.28)	0.49; % of children 12-59 months who are zinc deficient
Vitamin A supplementation	Child mortality due to diarrhea	Imdad et al 2011	0.70 (0.58, 0.86); 7 studies	High		NA
		Black et al 2013	0.70 (0.58, 0.86); 7 studies	High	RR attributed to vitamin A deficient fraction is 0.47 (0.34, 0.65)	0.53; % children 6-59 months who are vitamin A deficient
		Imdad et al 2017	0.88 (0.79, 0.98); 9 studies	High		NA
New intervention-outcome pairs						
Nutrition sensitive agriculture intervention	Appropriate complementary feeding	Margolies et al 2022	OR=1.64 (1.38, 1.94); 17 studies	Very low*	Starting Moderate; -2 for inconsistency; -1 for wide range of interventions included	No standard definition of the intervention
Zinc fortification	Diarrhea incidence	Black et al 2013	0.8 (0.73, 0.89); 13 studies	Low	Starting High; -1 for inconsistency; -1 because intervention is zinc supplementation<br>RR attributed to zinc deficient fraction is 0.35 (0.23, 0.52)	0.65; % of children 12-59 months who are zinc deficient
Zinc fortification	Pneumonia incidence	Black et al 2013	0.77 (0.65, 0.91); 3 studies	Moderate	Staring High; -1 because intervention is zinc supplementation<br>RR attributed to zinc deficient	0.52; % of children 12-59 months who are zinc deficient

					fraction is 0.48 (0.32, 0.72)	
Vitamin D supplementation	Pneumonia incidence	Martineau et al 2017	OR=0.96 (0.83, 1.10); 9 studies	Low	Starting High; -2 for including all ages and not limiting to LMICs	Do not include because evidence showed no impact
Vitamin D supplementation	Linear growth	Huey et al 2020	MD=62.7 (-0.37, 1.68); 3 studies	Low	Starting High; -1 for serious risk of bias; -1 for inconsistency	Do not include because evidence showed no impact
Zinc fortification	Stunting	Bhutta et al 2013	OR=0.90 (0.83, 0.96); 27 studies	Moderate	Staring High; -1 because intervention is zinc supplementation	Zinc fortification
Deworming	Weight	Thayer et al 2017	MD=-0.1 (-0.09, 0.29); 12 studies; MD=0.11 (-0.03-0.24); 14 studies	Very low	Starting Moderate; -1 for risk of bias; -1 for inconsistency	Do not include because the evidence showed no impact
Provision of SQ-LNS	Child anemia	Dewey et al 2021	0.79 (0.72, 0.87); 14 studies	Moderate	Starting High; -1 for inconsistency This review is SQ-LNS only	Do not include because lack of evidence on mortality impact
Multiple micronutrient powder	Child anemia	Suchdev et al 2020	0.82 (0.76, 0.9); 16 studies	Moderate	Starting High; -1 for inconsistency	Do not include because lack of evidence on mortality impact
Iron fortification	Child anemia	Keats et al 2019	0.61, (0.38, 0.96); 7 studies	Very low*	Starting Moderate; -2 for very serious inconsistency; -1 for wide range of interventions included and study population is <7 years	Do not include because lack of evidence on mortality impact

Zinc supplementation	Neonatal mortality due to sepsis	Irfan et al 2021	0.28 (0.12, 0.67); 2 studies	Very low*	Starting High; -1 for risk of bias; -2 for insufficient study participants; -1 for publication bias	Do not include because the study population was very small (n<300)
Zinc for treatment of sepsis	Neonatal mortality due to sepsis	Irfan et al 2021	0.49 (0.27, 0.87); 2 studies	Very Low	Starting High; -2 for insufficient study participants; -1 for publication bias	Do not include because the study population was very small (n<400)
Neonatal Vitamin A supplementation	Neonatal mortality due to diarrhea	Neonatal Vitamin A supplementation evidence group 2019	0.87 (0.80-0.94); 3 studies	Moderate	Starting High; -1 for only Asia	0.13; % pregnant women who are vitamin A deficient
Neonatal Vitamin A supplementation	Child <1 month mortality due to diarrhea	Neonatal Vitamin A supplementation evidence group 2019	0.87 (0.80-0.94); 3 studies	Moderate	Starting High; -1 for only Asia	0.13; % pregnant women who are vitamin A deficient
Zinc fortification	Child mortality due to diarrhea	Black et al 2013	0.81 (0.63, 1.03); 3 studies	Low	Starting High; -1 because intervention is zinc supplementation; -1 for wide CI RR attributed to zinc deficient fraction is 0.50 (0.27, 1.25)	0.50; % of children 12-59 months who are zinc deficient
Zinc fortification	Child mortality due to pneumonia	Black et al 2013	0.87 (0.67-1.14); 3 studies	Low	Starting High; -1 because intervention is zinc supplementation; -1 for wide CI RR attributed to zinc deficient fraction is 0.51 (0.20, 1.28)	0.49; % of children 12-59 months who are zinc deficient

Provision of SQ-LNS	Child mortality	Stewart 2020	0.82 (0.61-1.10); 15 studies	Moderate	Starting High; -1 for inappropriate intervention other than SQ-LNS included	The effect was insignificant when comparing to an active control arm and impact of SQ-LNS only was not available.

Very low*: the quality is downgraded to lower than very low
LiST-Lives Saved Tool, SQ-LNS-small quantity lipid-based nutrient supplementation, HAZ-height for age z-score, WHZ-weight for height z-score, MD-mean difference, IYCF-infant and young child feeding, SQ-LNS-small quantity lipid nutrient supplementation

Section Two: Description of the meta-analyses reviewed

Overview

This section provides a detailed description of systematic reviews and meta-analyses on nutrition interventions. For each intervention, we first list the outcomes reviewed to the right of the intervention. Next, we identify the meta-analysis for the intervention-outcome pair. Then we explain which meta-analysis is more appropriate to be used in LiST based on types of studies included; study settings; definition of intervention/comparison group; study population; and definition of the outcome. Finally, we determine the affected fraction, which is proportion of population who can benefit from the intervention based on study population in the meta-analysis and we also describe the data source for the affected fraction. A box with efficacy or odds ratio and affected fraction applied in the model is attached at the end of each included intervention.

Existing Interventions received by women of reproductive age (WRA) or pregnant women (PW)

Balanced energy protein supplementation (BEP)—stillbirth; SGA birth:

Two systematic reviews were compared for the impact of BEP (1, 2). Lassi et al excluded studies conducted in non-LMICs. Studies in Lassi et al compared BEP to standard of care (i.e. routine diet). Ota et al included studies conducted in non-LMICs, and any studies compared BEP to placebo or a comparison arm without BEP, meaning the comparison arm might receive other types of intervention. After checking studies included in Ota et al, we identified a relatively large study (Huybregts et al) with an active control arm where both intervention and control group received prenatal multiple micronutrients (3). We decided to use Lassi et al to estimate the effect size of BEP on stillbirth (RR=0.39, 95% CI 0.19, 0.80) and small for gestational age (RR=0.71, 95% CI 0.54-0.94) because 1) Not all pregnant women receive prenatal multiple micronutrients, In LiST, we want to estimate the isolated effect of BEP, 2) LiST is a modeling tool for LMICs and 3) Lassi et al included one more recent study (4).

To determine the affected fraction of the intervention, we checked the characteristics of the participants in the trials. Because the current affected fraction for BEP is women of reproductive age (WRA) with low BMI <18.5(5), we first checked the proportion of low BMI among the study population. In the five trials included, one reported mean BMI in the control=20.7 and intervention=21.3 group (6); one restricted to low BMI<18.5 (4); three studies did not have information on BMI (7-9). Besides BMI,

another common recruiting criterion, used by three trials are suboptimal nutrition status (6-8). Participants included in the trials were not necessarily women with low BMI. If we used low BMI as affected fraction, we might underestimate the impact of BEP. We think food insecurity as a cause for marginal nutrition status better describes the population that can benefit from BEP.

For food insecurity, we previously used the population living below $1.90 a day as a proxy in LiST. At the time, there were no data on prevalence of food insecurity that are available in enough countries for years 2000-2015 or that are the result of regular large-scale collection (5). Now the results of the Food Insecurity Experience Scale (FIES) were available for 77 countries from 2014-2018, reported in three-year averages (10). FEIS comprises eight questions that reflect self-reported behaviors and experiences associated with increasing difficulties in accessing food due to resource constraints(11). FEIS has three levels 1) food secure or mild food insecurity; 2) moderate food insecurity; 3) severe food insecurity. In LiST, we consider the prevalence of moderate or severe food insecurity as the prevalence of food insecurity. However, FEIS data were missing for several countries, including India and Pakistan which account for roughly 20% of the total LMICs population. To assess whether the percentage of the population living on <$1.90/day could be used for countries where FIES data were unavailable, we performed a correlation analysis in countries where both data were available. We found strong correlation between FIES and percentage of the population living on <$1.90/day (Pearson's correlation coefficient: 0.82), and therefore, use the percentage of the population living on <$1.90/day when FIES data are unavailable.

Outcome	Efficacy	Affected fraction	Reference
Stillbirth	0.61 (0.20, 0.81)	% of pregnant women who are food insecure	Lassi et al 2020
Small for gestational age birth	0.29 (0.06, 0.46)	% of pregnant women who are food insecure	Lassi et al 2020

Calcium supplementation—preterm birth; maternal mortality due to hypertensive disorder; pre-eclampsia:

We identified three systematic reviews for calcium supplementation during pregnancy (12-14). The most recent review by Oh et al excluded studies that collected data before 1995 (14). We think excluding older studies is inappropriate because the impact of calcium supplementation on various health outcomes is through biological pathways that hold true regardless of time. In addition, there were no big fluctuations in the prevalence of low calcium intake in LMICs over the past decade (15). Imdad et al only included studies with populations having low baseline low calcium intake and conducted in LMICs (12). A 2018 Cochrane review by Hofmeyr et al included studies with baseline adequate or low calcium intake participants and did not exclude studies conducted in non-LMICs (13).

Imdad et al and Hofmeyr et al reported results on preterm births. Imdad et al found a 12% reduction (RR=0.88, 95% CI 0.78, 0.99) among baseline low calcium intake population. In the Cochrane review, the pooled estimate for all studies was significant (RR=0.76; 95% CI 0.6, 0.97). But the subgroup analysis for baseline low calcium intake found an insignificant reduction in preterm (RR=0.81; 95% CI 0.64, 1.02). No consistent standard was used to classify low or adequate calcium intake, rather the classification was decided by individual trial authors (13). When we looked at the trend of low calcium intake in 10 LMICs with the largest total population, there were small fluctuations over time and low calcium intake was

predicted in approximately 70-99% of the population(15). Given the consistent evidence on the association between calcium supplementation and preterm, we decided to use pooled estimates from baseline low calcium intake studies from the Cochrane review to estimate the impact of calcium supplementation on preterm, and only apply the effect to the calcium deficient pregnant women in a country.

We did not identify any newer reviews that reported the impact of calcium supplementation on maternal mortality due to hypertensive disorder. We will keep using the estimates (RR=0.80, 95% CI 0.66, 0.98) from the Cochrane (13). It is noted that only one study was included for the outcome (16). But it is a relatively large study (n=8312) with a baseline low calcium diet population.

We did not currently have pre-eclampsia as a risk factor in LiST. In this round of review, we identified that both the 2018 Cochrane and Oh et al reported impact on pre-eclampsia (13, 14). Among the low baseline calcium intake population, the Cochrane found a 63% (RR=0.36, 95% CI 0.2-0.65, 8 studies) reduction and Oh et al found a 55% (RR=0.45, 95% CI 0.19-1.06, 4 studies) reduction in preeclampsia. Oh et al excluded studies that collected data before 1995 and with a smaller number of trials than included in the Cochrane found an insignificant reduction. We do not believe that it is appropriate to exclude older trials in this case. However, given the complex association between calcium supplementation, pre-eclampsia, and preterm birth, we decided not to add pre-eclampsia as a risk factor in LiST for now.

Since the participants in the trials have baseline low calcium intake, the affected fraction in LiST should also be percent of pregnant women with low calcium intake. We previously used the percent of population living under $1.90 a day as proxy due to lack of data availability on calcium intake. Now the country-specific prevalence of inadequate calcium intake has been estimated based on national food balance sheet data, UN population data, multiple food composition tables, and nutrient intake and requirement estimates(15). The data are available for all countries for years between 1961 to 2011. We will use these estimates for the affected fraction benefiting from calcium intake.

Outcome	Efficacy	Affected fraction	Reference
Preterm	0.19 (-0.02, 0.36)	% of pregnant women who are calcium deficient	Hofmeyr et al 2018
Maternal mortality due to hypertensive disorder	0.2 (0.02, 0.34)	% of pregnant women who are calcium deficient	Hofmeyr et al 2018

Iron with or without folic acid supplementation or multiple micronutrient supplementation during pregnancy and anemia—maternal anemia:

We identified two systematic reviews on iron supplementation with or without folic acid (14, 17). A 2015 Cochrane review found that compared to placebo, iron supplementation with or without folic acid reduced maternal anemia at term by 70% (RR=0.30; 95% CI 0.19-0.46, 14 trials) and iron-deficiency anemia at term by 67% (RR=0.33; 95% CI 0.16-0.69, 5 trials). A more recent systematic review—Oh et al found that compared to placebo, iron supplementation with or without folic acid reduced maternal anemia by 47% (RR=0.53; 95% CI 0.43, 6 trials). Oh et al did not report results for iron-deficiency anemia. The major difference between two reviews is that Oh et al excluded studies that collected data before 1995 or studies conducted in non-LMICs. Because it is not appropriate to exclude older studies, we decided to use pooled estimates on maternal anemia from 2015 Cochrane review as our reference. We changed the outcome from iron deficiency anemia to all anemia because 1) anemia is a greater and

more important public health concern in LMICs 2) there are not good country-specific prevalence trend data for iron-deficiency anemia. It is noted that studies have found no additional benefits on maternal anemia when comparing MMN to IFA (14, 18). Therefore, in LiST, we apply the same effect size to MMN and IFA. Since

Previous studies (17, 19) have also concluded that iron supplementation with or without folic acid does not have any impact on the risk of preterm births, (RR=0.85, 95% CI 0.67-1.08) or SGA (RR=0.93, 95% CI 0.84-1.03).

Previously we applied the impact of IFA to all pregnant women. Majority of the trials in the Cochrane review were conducted in LMICs, therefore we assumed the study population had low iron intake from their usual diet. We changed the affected fraction to percent of pregnant women who are iron deficient. The data for the affected fraction is based on national food balanced sheet (15).

Outcome	Efficacy	Affected fraction	Reference
Maternal Anemia	0.7 (0.54, 0.81)	% of pregnant women who are iron deficient	Pena-Rosa et al 2015

Multiple micronutrient supplementation during pregnancy (MMN)—preterm birth; SGA birth; stillbirth:

We identified three systematic reviews for the intervention (14, 18, 20). All three reviews reported results for SGA births and the findings were consistent. Oh et al only reported an overall effect (RR=0.93, 95% CI 0.88-0.98, 19 studies). Oh et al also excluded studies that collected data before 1995. But no study was excluded by this criterion. Two reviews performed subgroup analysis by BMI. In Smith et al, the overall effects (RR=0.94, 95% CI 0.90-0.98, 16 studies) and subgroup analysis by BMI<18.5 (RR=1.00, 95% CI 0.96-1.03, 16 studies) and BMI ≥18.5 (RR=0.97, 95% CI 0.95-0.99, 16 studies) were reported. In Keats et al, the overall effects (RR=0.92, 95% CI 0.88-0.97, 17 studies) and subgroup analysis by BMI<20 (RR=1.00, 95% CI 0.92-1.08, 3 studies) and BMI≥20 (RR=0.88, 95% CI 0.83-0.93, 14 studies) were reported. Both reviews found that MMN have little to no effect on SGA for women with lower BMI. The finding is a bit counterintuitive, but it is possible that for women with lower BMI, MMN is not sufficient to have an effect on SGA without improving energy and protein intake. Given the multiple functions of micronutrients, we still believe that women with lower BMI could benefit from MMN. Therefore we decided to apply the same effect size to all WRA. Since the overall effects were similar in three reviews, we decided to use Oh et al because it is the most recent systematic review.

To be consistent, we also decided to remove the stratification by BMI for the other birth outcome—preterm birth. The same three systematic reviews reported results on preterm (14, 18, 20). Smith et al found a 7% reduction (RR=0.93, 95% CI 0.87-0.98, 16 studies) in preterm births. Keats et al found a borderline insignificant reduction (RR=0.95, 95% CI 0.9-1.0, 18 studies) in preterm. Oh et al found an insignificant reduction (RR=0.96, 95% CI 0.91-1.01, 29 studies). Oh et al included an inappropriate study that compared calcium plus IFA supplementation to IFA supplementation (14). And the study is a relatively large study (n>8,000). In additional, a number of additional studies included in Oh et al were RCTs that examined the impact of one single micronutrient like zinc or vitamin D and IFA tablets were provided to both groups. Although the intervention did meet the minimum definition of MMN (at least 3 different micronutrients), they are less representative of MMN supplementation provided by various

programs. If we use the subgroup analysis for >4 micronutrients in Oh et al, a relatively large study with large effect size was excluded in the meta-analysis (21). We decided that pooled estimates from Oh et al was not appropriate to estimate the efficacy of MMS on preterm birth. Between Smith et al and Keats et al, the findings were similar, and we decided to use pooled estimates from Keats et al because it included two additional studies.

The same three systematic reviews also looked at stillbirth (14, 18, 20). In the fixed effects model, Smith et al concluded an 8% reduction (RR=0.92, 95% CI 0.86, 0.99; I^2=42%; 16 studies) in the risk of stillbirth. In the random effects model, Smith et al concluded an insignificant 3% reduction (RR=0.97, 95% CI 0.85, 1.11; 16 studies) in the risk of stillbirth. Keats et al found an insignificant 5% reduction (RR=0.95, 95% CI 0.86, 1.04; I^2=12%; 18 studies) in the risk of stillbirth using random effects model. Oh et al found a significant 9% reduction (RR=0.91, 95% CI 0.85, 0.98; 17 studies) for MMN trials contained 4 or more micronutrients using random effects model. The subgroup analysis for >4 micronutrients in Oh et al included similar set of studies as Keats et al and Smith et al, except one study which did not contribute much weight (21). We decided to use Oh et al as our reference for the association between MMN and stillbirth.

It is noted that all three reviews included studies comparing MMN to IFA. Given that IFA is not effective at reducing preterm, SGA or stillbirth, we concluded that IFA is an acceptable placebo control.

In both Oh et al and Keats et al, all pregnant women were eligible to receive MMN so our affected fraction for the intervention is all women of reproductive age.

Outcome	Efficacy	Affected fraction	Reference
Small for gestational age birth	0.07 (0.02, 0.12)	All pregnant women	Oh et al 2020
Preterm birth	0.05 (-0.01, 0.10)	All pregnant women	Keats et al 2019
Stillbirth	0.09 (0.02, 0.15)	All pregnant women	Oh et al 2020

Iron fortification—maternal anemia:

Currently we used a 2019 systematic review on large-scale fortification as our reference (22). We did not identify a newer review for the intervention. In the subgroup analysis, iron fortification reduced anemia prevalence among both non-pregnant women (RR=0.66; 95% CI 0.56-0.76, 9 studies) and pregnant women (RR=0.73; 95% CI 0.64-0.84, 3 studies). We used to apply the same effect size of 34% reduction in iron-deficiency anemia for all WRA. For the similar reason as iron supplementation, we decided to change the outcome to all anemias. And we apply different effect sizes for non-pregnant women and pregnant women to best reflect findings in the review. To our knowledge, no study has examined the combined effect of iron supplementation and fortification. In LiST, the overall effect of the two interventions will be the sum of the two individual effects.

It is noted that the types of studies included in the review varied and there was not too much information on the prevalence of fortification to reach the anemia benefits. Assuming the impact of the iron fortification acts through providing sufficient iron to meet the requirement for women of reproductive age, in LiST we defined the prevalence of iron fortification as percent of women 15-49 years old that receive iron food fortification (18 mg iron per day). The details on the data and method used to estimate the prevalence were published elsewhere (23).

Outcome	Efficacy	Affected fraction	Reference
Maternal anemia	0.34 (0.24, 0.44)	% of non-pregnant women of reproductive age who are iron deficient	Keats et al 2019
Maternal anemia	0.27 (0.16, 0.36)	% of pregnant women who are iron deficient	Keats et al 2019

Periconceptual folic acid supplementation/fortification—stillbirth due to neural tube defects; neonatal mortality due to neural tube defects; preterm birth; child mortality due to neural tube defects:

For periconceptual folic acid fortification and stillbirth, we did not find a more recent review than the one used previously, which included 11 before and after studies and found a 41% (RR=0.59, 95% CI 0.52, 0.68) reduction in neural tube defects (24). The same effect size was applied to intrapartum and antepartum stillbirth due to neural tube defects.

Keats et al review the association between periconceptual folic acid fortification and neonatal mortality due to neural tube defects. The review only included studies conducted in LMIC and large-scale fortification (≥1000 individuals per arm) (21). Both observational and experimental studies were included. The pooled effect (RR=0.59, 95% CI 0.49-0.70, 8 studies) is on reduction in neural tube defects prevalence and this is applied to neural tube defect deaths.

Since all the studies were conducted in LMICs, we assumed the study population were in some degree of folate deficiency. Therefore, the affected fraction for the intervention is percent of women reproductive age who are folate insufficient.

We also identified a 2018 meta-analysis of folic acid and risk of preterm birth (25). The review only included observational studies. The intervention was the highest category of folic acid supplementation or dietary folate intake. The comparison was the lowest category of folic acid supplementation or dietary folate intake. Information on exposure of interest was collected via food frequency questionnaires or interviews. The lowest category observed in the included studies ranged from 0 to 200 µg daily, and the highest category ranged from any folic acid or folic acid-containing supplements consumption to ⩾1000 µg daily. The outcome—PTB is defined as delivery at <37 weeks gestation.

A total of 14 cohort studies on folic acid supplementation were included for the meta-analysis and they found an inverse association (adjusted OR=0.90, 95% CI 0.86-0.95). In the subgroup analysis by initiation time of supplementation, only preconception folic acid supplementation reduces the odds of PTBs (OR=0.87, 95% CI 0.84-0.91, 8 studies). Three studies reported the overall risk of PTB in relation to dietary folate intake. When comparing the highest category to lowest category, the odds ratio is 0.68 (95% CI 0.55-0.84). Due to small number of studies, no subgroup analysis was performed.

Of the 8 included studies for preconception folic acid supplementation, 5 were conducted in high-income countries and 3 were conducted in upper-middle income countries. When we checked the adjusted covariates, none of the included studies adjusted for other preconception vitamin use. The largest study included in the meta-analysis, which accounted for about 67% weight, provided folic acid only pills (26). In summary we think there was weak evidence from the review showing an association between increased preconception folate intake and reduced PTB. Since there are few national programs

for folic acid supplementation before pregnancy, we decided to also model the impact via periconceptual folic acid fortification.

Since the pooled estimate was reported as OR, we need to convert the OR to RR. We looked at all the individual studies included in the meta-analysis and calculated the weighted preterm birth rate among non-folic acid users. Two out of eight studies did not report the preterm birth rate among the non-folic acid users. One was a study conducted in US (27). Since two other included studies were also conducted in US with a 7% and 9% PTB rate among non-folic acid users (28), we used the average 8% for the US study with missing PTB rate (27). The other study with missing PTB rate was conducted in the Netherlands. Since Netherland is a high-income country, we used a national estimate on spontaneous PTB rate (29). Overall we got a weighted PTB rate of 5% and used it to convert the OR to RR=0.88 (0.85, 0.91). The efficacy of periconceptual folic acid fortification and preterm birth is 0.12 and the affected fraction is percent of population who are folate deficient. The prevalence of folate deficiency is drawn from Beal et al 2017 (15).

No direct data on association between periconceptual folic acid and child mortality due to neural tube defects were available. But evidence has shown that certain proportion of neonates with neural tube defects can survive up to at least 1 year of age (30). To model the potential impact of folic acid fortification on child mortality due to neural tube defects, we need to have data on proportion of child mortality that are attributable to neural tube defects. Unfortunately, such data is not available right now. Therefore, we decided not to include the link between folic acid fortification and child mortality due to neural tube defects now.

Outcome	Efficacy	Affected fraction	Reference
Stillbirth due to neural tube defects	0.41 (0.32, 0.48)	% of non-pregnant women of reproductive age who are folate insufficient	Imdad et al 2011
Neonatal mortality due to neural rube defects	0.41 (0.30, 0.51)	% of non-pregnant women of reproductive age who are folate insufficient	Keats et al 2019
Preterm birth	0.12 (0.09, 0.15)	% of non-pregnant women of reproductive age who are folate insufficient	Li et al 2019

Potential new interventions for women of reproductive age or pregnant women

Vitamin D supplementation for pregnant women—preterm births; pre-eclampsia:

A recent Cochrane review examined vitamin D supplementation and its health outcomes(31). Trials included in the review differed in frequency of supplementation, ranging from daily, weekly, monthly to single dose. Averaged daily dose of vitamin D also varied in the included trials. Vitamin D supplementation alone was found to be effective at reducing pre-eclampsia (RR=0.48; 95% CI 0.3-0.79, 4 trials). Vitamin D alone does not reduce risk of preterm (RR=0.66; 95% CI 0.34-1.3, 7 trials). The review also looked at vitamin D + low dose calcium vs placebo, which is effective at reducing preeclampsia but had negative impact on preterm (RR=1.52; 95% CI 1.01-2.28, 4 trials). No information is available for vitamin D and high dose calcium (>1g/d), which is an intervention for pregnant women in LiST. Given the potential increased risk on preterm with the combination of vitamin D and calcium, we decided not to include vitamin D for pregnant women in LiST.

Stop smoking education for pregnant women who smoke—preterm birth:

There was evidence showing that stop smoking education might reduce preterm among pregnant women who smoke (32). The review included 17 trials, all conducted in high-income settings. Pregnant women who were current or recent smokers were recruited but the criteria used to classify a 'smoker' varied substantially between trials. The overall pooled estimates find a small but not significant reduction in preterm (RR=0.93; 95% CI 0.77-1.11). The review did find a significant reduction in low birth weight (RR=0.83; 95% CI 0.72-0.94). The significant pooled estimate is probably by reducing small-for-gestational-age newborns but the impact on SGA was not reported in the meta-analysis. Since there was no evidence from LMICs and LiST needs an effect on preterm or SGA, we decided not to include the intervention.

Deworming for pregnant women—maternal anemia:

A recent Cochrane review assessed the effect of mass deworming on maternal anemia (33). The study population were pregnant women in second trimester of pregnancy. And the study settings were antenatal clinics covering 6 LMICs. When comparing anthelminthics to placebo or no anthelminthics control, there is little to no reduction in maternal anemia in third trimester (RR=0.85; 95% CI 0.72-1.00, 5 RCTs; low certainty). Most of the trials (n=4) in the review provided IFA tablet to both groups. Because the effect is marginally significant and with low certainty, we decided not to include this intervention in LiST.

Maternal thiamine supplementation—neonatal mortality:

Evidence showing increased breast milk thiamine concentration with supplementation in thiamine deficient populations(34). But there were no direct data for the infant-related outcomes. Due to the insufficient data, we decided not to include this intervention.

Zinc fortification—preterm birth:

Recent meta-analysis found that consumption of zinc fortified food increased plasma/serum zinc concentration, with a corresponding decrease in the prevalence of zinc deficiency (35). Since there were not enough studies on the impact of zinc fortification on outcomes like preterm births, the potential impact could be estimated based on the effect of zinc supplementation.

A recent Cochrane review suggests that zinc supplementation during pregnancy may result in little or no difference in reducing preterm (RR=0.87; 95% CI 0.74-1.03, 21 studies)(36). The analysis did not distinguish between population of low zinc and nutrition and those of normal zinc and nutrition, which is different than the previously published 2015 Cochrane. In the 2015 Cochrane review, the subgroup analysis for women with "low zinc or nutrition" found a treatment effect of RR=0.87 (0.77-0.98)(37). The low zinc or nutrition is defined as women in an area where there is some zinc deficiency, not strictly women who have been determined to be zinc deficient.

Among the five new studies included in the more recent Cochrane, only one specified that the participants had baseline low level of zinc serum. The other four did not provide information on baseline zinc status. But the countries where the four studies were conducted all had a prevalence of low zinc intake greater than 30% (15). Given that supplementation trials showed little to no difference in reducing preterm birth and more research is needed to understand if zinc fortification performed the same way as supplementation, we are not confident to extrapolate the potential impact to zinc fortification.

Omega-3 fatty acid supplementation—preterm births:

We identified a 2018 Cochrane review that looked at omega-3 fatty acid for pregnant women and various perinatal outcomes (38). Only RCTs were included in the review. The review defined the intervention as any forms, types, or dose of omega-3 fatty acid with or without co-interventions. And the control was defined as placebo or no omega 3. All pregnant women were eligible.

Preterm birth <37 weeks (RR=0.89, 95% CI 0.81-0.97; 26 RCTs) and early preterm birth <34 weeks (RR=0.58; 95% CI 0.44-0.77, 9 RCTs) were both lower in women who received omega-3 compared with no omega-3. The intervention had little to no impact in SGA (RR=1.01; 95% CI 0.90-1.13) and pre-eclampsia (RR=0.84; 95% CI 0.69-1.01). The review also found that omega-3 fatty acid might increases prolonged gestation >42 weeks (RR=1.61, 95% CI 1.11-2.33). Despite the 16 trials (7880 participants) included the confidence interval for stillbirth was still wide (RR=0.94; 95% CI 0.62-1.42).

In subgroup analysis by types of interventions, omega-3 supplements only showed an insignificant reduction in PTB<37 weeks (RR=0.90, 95% CI 0.8-1.01, 18 RCTs) but a significant reduction in early PTB<34 weeks (RR=0.62, 95% CI 0.46-0.82). For studies that provided omega-3 supplements and other agents, the other agents were not always provided for the control group as well. Since some of the studies used a co-intervention like multiple micronutrients which can also reduce risk of PTB, the effect sizes of omega-3 from these studies might be driven by MMS.

In addition, majority of the included studies were conducted in high- income countries (22 out of 26). Causes of PTB are multifactorial, the high-income settings might not reflect the similar multifactorial causes of PTB birth in LMICs. For example maternal infections like malaria and various micronutrient deficiency are also causes of PTB but the prevalence of these risk factors might vary a lot in HICs vs LMICs.

In summary, there is weak evidence showing the potential impact of the omega-3 fatty acid supplementation on PTB, but little of this data is from LMICs. Moreover there is evidence of the adverse impact of omega 3 on prolonged gestation>42 weeks and confidence interval for stillbirth is too wide to rule out harm. Future studies in LMIC must be conducted with extra caution on the potential side effects of the intervention. We recommended not to include this intervention in LiST.

Existing Interventions for infants and children 0-59 months

Infant and young child feeding (IYCF) education—early initiation of breastfeeding; exclusive breastfeeding; continued breastfeeding

IYCF education can promote early initiation of breastfeeding within 1 hour of birth, exclusive breastfeeding (EBF) for 1-6 months and continued breastfeeding for 6-23 months. In two recent reviews the major difference for the types of studies included is that observational studies were included in Sinha et al but not Lassi et al (39, 40). Both reviews provided subgroup analysis by delivery setting: health system and home/community. Sinha et al was clearer with the categorization in the review, providing examples of delivery settings in each category. And they also included an additional category—combined, where interventions were delivered concurrently in multiple settings. Lassi et al briefly mentioned the categorization which was based on the person who delivered the intervention. For example interventions delivered by doctors or nurses were considered facility based.

Sinha provided clear definition of various breastfeeding related outcomes. For example, if a study examined EBF at multiple time points, the longest time data were used in the meta-analysis. Lassi et al did not provide any specific definition of the outcomes. In addition, for EBF we have two subgroups (<1 months and 1-5months) in LiST. Sinha provided subgroup analysis for the two age ranges. Lassi et al only reported results on EBF<3 months and EBF <6 months. They also did not report analysis for continued breastfeeding.

In Sinha et al, unadjusted and adjusted OR were combined in the meta-analysis. In Lassi et al, the overall quality of outcomes was graded low or very low based on GRADE method due to serious inconsistency across studies. For each outcome, Sinha et al included more studies in the meta-analysis. Based on the information collected, we think Sinha et al had a better study design and provided more appropriate effect sizes needed for LiST. Therefore we decided to keep using Sinha et al as our reference. The affected fractions correspond to the different age ranges (<1 months, 1-5months, or 6-23 months).

Outcome	Odds ratio	Affected fraction	Reference
Early initiation of breastfeeding	Health system: 1.82 (1.32, 2.50)<br>Home/community: 3.38 (1.97, 5.90)<br>Combined setting: 4.96 (2.88, 8.54)	Infants <1 month	Sinha et al 2017
Exclusive breastfeeding <1 month	Health system: 2.03 (1.33, 3.10)<br>Home/community: 2.17 (1.84, 2.56)<br>Combined setting: 2.33 (0.85, 6.45)	Infants <1 month	Sinha et al 2017
Exclusive breastfeeding 1-6 months	Health system: 3.07 (2.09, 4.52)<br>Home/community: 2.48 (1.99, 3.09)<br>Combined setting: 6.8 (3.75, 12.33)	Infants 1-6 months	Sinha et al 2017
Continued breastfeeding 6-23 months	Health system: 1.42 (0.88, 2.28)<br>Home/community: no estimates<br>Combined setting: 1.42	Infants 6-23 months	Sinha et al 2017

Zinc for treatment of diarrhea—neonatal mortality due to diarrhea; child mortality due to diarrhea:

We identified two systematic reviews on the topic (41, 42). Walker & Black et al identified high evidence of morbidity reduction based on 2 cluster RCT and >300 hospitalization but low evidence for mortality reduction. The Cochrane review did not report results for diarrhea hospitalizations. Among the four studies included for the meta-analysis on mortality, there were only 3 deaths in zinc group and 8 deaths in placebo group. It is not appropriate to estimate RR of death based on the few deaths.

In LiST, we are comparing children experiencing diarrhea and received zinc vs those who did not. We first assumed that a severe diarrhea incidence would require hospitalization in LMICs. And then we assumed that a severe diarrhea is a proxy for mortality. Therefore we decided to use diarrhea hospitalization to estimate the effect on mortality, using numbers from Walker & Black et al.

Outcome	Efficacy	Affected fraction	Reference
Neonatal mortality due to diarrhea	0.23 (0.15, 0.31)	Infants <1 month with diarrhea	Walker & Black et al 2010
Child mortality due to diarrhea	0.23 (0.15, 0.31)	Children 6-59 months with diarrhea	Walker & Black et al 2010

Complementary feeding education only—stunting:

We identified two systematic reviews that examined complementary feeding education (40, 43). Both studies found that complementary feeding education only is only beneficial in a food secure population, but not in a food insecure population. For food insecure population, the suboptimal diet is mainly due to restricted resources therefore education only is not sufficient to improve the diet. Only one study was included in both reviews. In Panjwani et al, food secure is classified primarily based on narrative of baseline population characteristics; secondary approach is to use World Bank national data where upper middle-income is considered food secure. Lassi et al classified level of food security using information in the full texts of the article. In addition, Panjwani et al used WHO growth standard in the meta-analysis. Because Panjwani et al used a clearer definition of food security and WHO growth standard, we decided to keep using Panjwani et al as our reference. We will also update our affected fraction with data from food insecurity experience survey.

Outcome	Odds ratio	Affected fraction	Reference
Stunting	1.3 (1.1, 1.5)	Percent of children 6-23 months who are food secure	Panjwani et al 2017

Provision of appropriate fortified complementary foods—stunting; wasting; child mortality; child anemia:

We identified three systematic reviews for provision of complementary foods (40, 43, 44). Panjwani et al and Lassi et al included studies providing any types of complementary food. Panjwani et al and Lassi et al also accepted both RCTs and non-randomized studies such as controlled before-after studies. Dewey et al is an individual participant data meta-analysis of RCTs for small-quantity lipid-based nutrient supplements (SQ-LNS) and child growth. All three reviews compared one type of complementary food to a control group without any food supplement for children. The pooled estimates on child growth were similar across three reviews.

We wanted to isolate the effect of non-LNS complementary food, but to our knowledge, there is no review focused on non-LNS only. Therefore we conducted a de novo meta-analysis based on the studies included in Panjwani et al and Lassi et al. Within the non-LNS category, we broke it further down to local food and prepared non-LNS food. Local food is defined as food mixture without fortification using locally available ingredients or other un-mixed local food, regardless of caloric content. Prepared non-LNS is defined as food-based matrix that is not LNS with fortified multiple micronutrients. It was noted that all prepared non-LNS contains more than ~125 kcal. We excluded the intervention arms that provided

medium-quantity-LNS (45, 46). Two studies included in Lassi et al is not appropriate. One study did not provide any types of complementary food (47). One study provided other food supplement for children in the control group (48).

By pooling the effect size for the subcategories of complementary food we want to 1) determine if SQ-LNS is more effective than other types of complementary food in improving growth outcomes 2) check if local food without any fortification can improve growth outcomes. We decided to use absolute mean difference (MD) between intervention and control groups in change in height-for-age z-score (HAZ) and weight-for-height z-score (WHZ) from baseline to end line as our effect size. Because all three reviews reported these outcomes, and more studies were included for the outcomes. Since Panjwani et al applied stricter criteria where studies with high attrition rate or insufficient sample size were excluded, we conducted two sets of meta-analysis—one with stricter inclusion criteria and one with looser inclusion criteria.

Two studies were excluded under the stricter criteria, but since the two studies were relatively small, the exclusion did not impact the overall pooled estimates, see section four (49, 50). In random-effects model, the overall pooled estimate on WHZ (MD=0.07, 95% CI 0.03-0.11, 18 studies) was similar to the findings in Panjwani et al (MD =0.07, 95% CI 0.03-0.12, 7 studies). In the subgroup analysis, both local food (MD=0.07, 95% CI -0.72-0.87, 2 studies) and prepared non-LNS (MD=0.01, 95% CI -0.09-0.87, 2 studies) were not effective at reducing WHZ.

For HAZ, the overall pooled estimates (MD=0.14, 95% CI 0.08-0.19, 20 studies) in random effects model was also similar to the findings in Panjwani et al (MD=0.10, 95% CI 0.03-0.17, 7 studies). In the subgroup analysis, both local food (MD=0.34, 95% CI -0.20-0.78, 3 studies) and prepared non-LNS (MD=0.04, 95% CI -0.12-0.21, 3 studies) were not effective at reducing HAZ.

The results were generally similar in random-effects model and fixed effects model. The only significant difference is the subgroup analysis for local food. In the fixed effects model, local food reduced WHZ (MD=0.33; 0.12-0.55) and HAZ (0.50; 0.29-0.71). Given the high heterogeneity among studies on local food, we think random-effects model is more appropriate.

Without searching for other studies of prepare non-LNS or local food, we decided to rename the intervention “Provision of appropriate fortified complementary food”. The definition of the intervention is providing fortified and nutritious food. And we will make a note that the effect size is based on SQ-LNS trials. We used the same method described in Panjwani et al to convert MD to OR (43). The affected fraction for the intervention is percent of population who are food insecure. And the prevalence data are from the food insecurity experience scale and poverty headcount ratio at $1.9 a day.

A recent meta-analysis also looked at SQ-LNS and child mortality (51). The paper included RCTs, comparing small- and medium-quantity LNS with non-LNS controls. Small- and medium- quantity LNS was defined as <500 kcal/day. The non-LNS arm did not receive any other types of child supplementation, such as multiple micronutrient powder or other fortified blended food. The review also excluded trials with maternal LNS supplementation or trials focused primarily on treatment of malnutrition. The outcome is all cause child mortality after 6 months. The risk of mortality was lower in the LNS arms than in the non-LNS control arms (RR=0.73, 95% CI 0.59-0.89, 13 studies). But when comparing multicomponent arms with LNS groups and comparison groups that contained all the same components except LNS, the effect estimate was attenuated (RR=0.82, 95% CI 0.61-1.10, 15 studies).

Most of the studies (n=12) provided SQ-LNS. Given that 1) comparing to active control arm, the effect was attenuated; 2) the study did not report cause-specific mortality; 3) there was no subgroup analysis for SQ-LNS only, we decided to not add the link between provision of complementary food and child mortality.

We identified a IPD meta-analysis on SQ-LNS and child anemia (52). Only RCTs conducted in LMICs are included in the meta-analysis. The intervention group received SQ-LNS with or without a co-intervention. The control group received placebo or an intervention without any types of LNS or other child supplement. Child anemia is defined as Hb<110 g/L. All the trials included in the review were conducted among children 6-24 months. SQ-LNS increased hemoglobin concentration (MD=2.77 g/L; 95% CI 2.31, 3.23 g/L) and reduced the prevalence of anemia (PR=0.84, 95% CI 0.81-0.87, 14 trials). Since SQ-LNS contains certain amount of iron, it is logical to see a reduction in child anemia.

However, it is complex to include child anemia into the LiST model. Child anemia determined by hemoglobin level should be interpreted as a distribution, meaning that an intervention can improve hemoglobin level but if an individual has a very low baseline hemoglobin level, the improvement might not be sufficient to make the individual non-anemic. To include child anemia as a risk factor in LiST, we need to have some baseline information on the hemoglobin distribution among children in different countries. But such detailed country-level data was not available right now. Another concern is that LiST is a mortality-focused model, meaning all the risk factors are linked to mortality or another risk factor. However, currently we do not have evidence on the connection between child anemia and mortality. Therefore, we decided that we will not include the impact of SQ-LNS on child anemia in the model until further data and evidence on child anemia become available.

Outcome	Odds Ratio	Affected fraction	Reference
Stunting	1.66 (1.48, 1.86)	Percent of children 6-23 months who are food insecure and received complementary food	Dewey et al 2021
Stunting	1.92 (1.64, 2.28)	Percent of children 6-23 months who are food insecure and did not receive complementary food	Dewey et al 2021
Wasting	1.5	Percent of children 6-23 months who are food insecure and received complementary food	Dewey et al 2021
Wasting	1.64	Percent of children 6-23 months who are food insecure and did not receive complementary food	Dewey et al 2021

Vitamin A supplementation-diarrhea incidence; child mortality due to diarrhea:

We identified three meta-analyses for vitamin A supplementation and diarrhea incidence (53-55). Tam et al excluded the studies published before 1995 when many Vitamin A trials were conducted and the impact was not significant (RR=0.97, 95% 0.83-1.14, 7 studies) (55). The 2017 Cochrane review (RR=0.85, 95% CI 0.82-0.87, 14 studies) included two more recent studies than Black et al (unadjusted RR=0.72, 95% CI 0.72-1.00, 12 studies) (56, 57). It was important to note that Black et al attributed the overall effect to the vitamin A deficient fraction in the trial populations. Using the protective RRs from RCTs and the prevalence of low serum retinol, Black et al calculated adjusted RR=0.62 (0.44-0.87) for diarrhea incidence. We think it is important to conduct this adjustment so that our estimated impact of vitamin A supplementation included the time varying prevalence of vitamin A deficiency. Therefore, we decided to

keep using adjusted RR from Black et al and apply to the present prevalence of vitamin A deficient children.

We identified three meta-analyses for vitamin A supplementation and child mortality due to diarrhea (53, 54, 58). The 2011 Cochrane review and Black et al included the same set of trials. But in Black et al, with the assumption that all the effects were in the subset of the trial participants with low serum retinol, the adjusted RR was calculated. The newer Cochrane review added the DEVTA trial published in 2013, where it found little to no impact on child mortality due to diarrhea (59). DEVTA trial has received various criticisms since publication (60-62). We decided to keep using adjusted RR from Black et al and apply to the present prevalence of vitamin A deficient children.

The affected fraction for the intervention is percent of children who are vitamin A deficient. We used country-specific prevalence of inadequate vitamin A intake as proxy for prevalence of vitamin A deficiency. Data are available for all countries included in LiST for years between 1961 to 2011 (15). The data were estimated based on national food balance sheet data, UN population data, multiple food composition tables, and nutrients intake and requirements data. We were aware that other studies have also reported prevalence of low serum retinol in LMICs (63). But the data were not available for time trends.

Outcome	Efficacy	Affected fraction	Reference
Diarrhea incidence	0.38 (0.13, 0.56)	Percent of children 6-59 months who are vitamin A deficient	Black et al 2013
Child mortality due to diarrhea	0.53 (0.35, 0.66)	Percent of children 6-59 months who are vitamin A deficient	Black et al 2013

Zinc supplementation—diarrhea incidence; pneumonia incidence; child mortality due to diarrhea; child mortality due to pneumonia; stunting:

We identified three reviews on zinc supplementation for children and disease incidence—diarrhea and pneumonia (53, 55, 64). The RR for diarrhea incidence and pneumonia incidence were consistent across three reviews. The same assumption adjustment for vitamin A supplementation were also applied for zinc supplementation (53). Since we want to account for time varying zinc-deficiency, we decided to use Black et al as our reference.

Two reviews looked at zinc supplementation and cause-specific child mortality—mortality due to diarrhea and mortality due to pneumonia (53, 64). Three studies were included in both reviews. Therefore, the pooled estimates were consistent within the two reviews. Black et al also reported adjusted RR that attributed the overall effect to the zinc deficient fraction. Another older review found significant impact on all-cause mortality among children 12-59 months (65). Since the impact for all-cause mortality is significant and there are significant effects on diarrhea and pneumonia incidence, there is increased plausibility of the mortality effects. We decided to include the impact.

We identified two reviews on zinc supplementation and stunting (55, 66). Bhutta et al reanalyzed the studies included in the subgroup analysis for children 1-4 years old in the Cochrane review (67). By converting standard mean difference in height (SMD=0.11, 95% CI 0.04-0.18, 27 studies) to odds ratio, they found a small but significant impact on stunting (OR=0.90; 95% CI 0.83-0.96, 27 studies). Tam et al included studies conducted in healthy children 1 months to 5 years of age living in LMICs. Tam et al

found insignificant impact on height (MD=0.04, 95% CI -0.12-0.20, 18 studies) and stunting (RR=1.00; 95% CI 0.89-1.14, 6 studies). The major difference between the two reviews is the age range of the study population. Eight studies included in Tam et al included infants 1-12 months.

The Cochrane review found that zinc supplementation has negative impact on linear growth in children 6-12 months (SMD=-0.26; 95% CI -0.33, -0.19)(67). There were three new studies included in the more recent systematic review, but their study population also included 6-12 months infants. We think that different age range inclusion explained the insignificant findings in Tam et al. Since we have the estimate for prevalence of zinc deficiency among children 1-4 years, we will keep the impact of zinc supplementation on stunting and use the pooled estimates from Bhutta et al.

The affected fraction for the intervention is percent of children 1-4 years who are zinc deficient. Currently we use prevalence of low zinc intake as a proxy to estimate the prevalence of zinc deficiency (68). But evidence has shown that this value may underestimate the prevalence of zinc deficiency as measured by plasma or serum zinc concentration (69). But the prevalence of zinc deficiency based on plasma/serum zinc is only available for 19 countries. When comparing percentage of low plasma/serum zinc with low zinc intake, the results were not consistent, so it is hard to apply a formula for adjustment. More work is need for the better data on prevalence of zinc deficiency.

Outcome	Efficacy	Affected fraction	Reference
Diarrhea incidence	0.65 (0.48, 0.77	Percent of children 12-59 months who are zinc deficient	Black et al 2013
Pneumonia incidence	0.52 (0.28, 0.68)	Percent of children 12-59 months who are zinc deficient	Black et al 2013
Child mortality due to diarrhea	0.50 (-0.25, 0.73	Percent of children 12-59 months who are zinc deficient	Black et al 2013
Child mortality due to pneumonia	0.49 (-0.28, 0.20)	Percent of children 12-59 months who are zinc deficient	Black et al 2013
Stunting	OR=1.11 (1.04, 1.20)	Percent of children 12-59 months who are zinc deficient and did not receive zinc supplementation	Bhutta et al 2013

Potential new interventions for infants and children 0-59 months

Deworming for children 1-59 months—wasting:

Systematic reviews of deworming were often conducted for children under 16. A 2017 literature review identified randomized and quasi-randomized trials for meta-analysis on outcomes assessed in populations that primarily contained children younger than 5 years (70). They identified two systematic reviews and then conducted a meta-analysis using appropriate trials included in the two reviews. Two new meta-analyses based on studies for children younger than 5 years did not find significant effect of deworming on weight—Taylor-Robinson et al (MD=0.10; 95% CI -0.09-0.29) and Croke et al 2016 (MD=0.11; 95% CI -0.03-0.24)(71, 72). Since the meta-analysis showed no impact on weight or other growth outcomes, we decided not to include this intervention in LiST.

Vitamin D for children 1-59 months—pneumonia incidence; stunting:

We identified an IPD meta-analysis of 25 RCTs on vitamin D and respiratory infection (73). While there is an effect on all acute respiratory infection with weekly or daily dosing (0.81; 0.72-0.91), a possible effect

on upper respiratory infection (URTI, RR=0.88; 0.78-1.00), there was no effect on lower respiratory infection (LRTI RR=0.98; 0.75-1.28). LiST does not include URTI. A recent Cochrane review also examined vitamin D and growth outcomes for children under five years of age (74). Compared to placebo, oral vitamin D (200 to 2000 IU daily), does not improve linear growth (MD=0.66; 95% CI -0.37-1.68, 3 RCTs). Given the evidence from two systematic reviews, we decided not to include the intervention.

Prophylactic antibiotics for children 1-59 months—stunting:

To our knowledge, there were no systematic review conducted for the intervention. Due to the insufficient data, we will not include this intervention.

Nutrition sensitive agriculture intervention—appropriate complementary feeding:

We did not identify any studies that looked at the direct impact of nutrition sensitive interventions and growth outcomes. A recently published meta-analysis found potential impact on diet diversity score and minimum diet diversity in children 6-59 months (75). However, the definition of nutrition sensitive intervention is not clear, and the actual interventions varied a lot across studies. Since the standard of the intervention is vague and the impact pathway is more complex than fixing micronutrient status, we decided that it is not appropriate to include the intervention in LiST.

Zinc fortification—diarrhea incidence; pneumonia incidence; child mortality due to diarrhea; child mortality due to pneumonia; stunting:

Recent meta-analysis found that consumption of zinc fortified food increased plasma/serum zinc concentration, with a corresponding decrease in the prevalence of zinc deficiency (35). Since there were not enough studies on the impact of zinc fortification on outcomes like diarrhea incidence, pneumonia incidence, and stunting, the potential impact could be estimated based on the effect of zinc supplementation.

We extrapolated the estimates of effect size based on zinc supplementation trials to zinc fortification (53, 66).

Outcome	Efficacy	Affected fraction	Reference
Diarrhea incidence	0.65 (0.48, 0.77)	Percent of children 12-59 months who are zinc deficient	Black et al 2013
Pneumonia incidence	0.52 (0.28, 0.68)	Percent of children 12-59 months who are zinc deficient	Black et al 2013
Child mortality due to diarrhea	0.50 (-0.25, 0.73)	Percent of children 12-59 months who are zinc deficient	Black et al 2013
Child mortality due to pneumonia	0.49 (-0.28, 0.20)	Percent of children 12-59 months who are zinc deficient	Black et al 2013
Stunting	OR=1.11 (1.04, 1.20)	Percent of children 12-59 months who are zinc deficient and did not receive zinc supplementation	Bhutta et al 2013

Iron fortification—child anemia:

We identified a systematic review on iron fortification and child anemia (22). Both observational studies that evaluate national/subnational fortification program and large scale (n>1000 per arm) randomized or quasi-randomized controlled trials were included in the review. All the included studies were

conducted in LMICs settings. The fortification standard in the intervention group ranged from 2.2 mg/100g to 29.6 mg/100g. The control group was either a non-fortified group or a pre-fortification population. The review looked at children less than 7 years old. Iron fortification was found to reduce anemia (RR=0.61; 95% CI 0.38-0.96, 7 studies). The intervention might also improve hemoglobin concentration (SMD=0.30; 95% CI -0.05, 0.66, 6 studies). Since iron fortification is likely to increase children's iron intake, it is logical to observe potential improvement in hemoglobin concentration and anemia. But there are lack of connection between child anemia and mortality and lack of data availability on hemoglobin distribution among children. Therefore we decided not to add the link between iron fortification and child anemia in LiST until further data and evidence on child anemia become available.

Multiple micronutrient powder for children—child anemia:

We identified two Cochrane reviews for multiple micronutrient powder (76, 77). Both reviews only included RCTs. The intervention was addition of powders containing vitamins and minerals (at least iron, zinc, and vitamin A) to semi-solid food immediately before consumption. The intervention is also known as point of use fortification. The control was placebo or an intervention without iron-containing supplements. The target population in the two reviews were different: one for children ages 6 to 23 months and the other for children 2-12 years with subgroup analysis for 2-5 years. The intervention reduced the risk of anemia by 18% (RR 0.82, 95% CI 0.76 to 0.90; 16 studies; 9927 children; moderate-certainty evidence) and increased hemoglobin concentrations (MD 2.74 g/L, 95% CI 1.95 to 3.53; 20 studies; 10,509 children; low-certainty evidence) for children under 2 years (76). The intervention reduced the risk of anemia by 36% (RR 0.64, 95% CI 0.44 to 0.93; 6 studies; 1706 children) and increased hemoglobin level (MD=2.02, 95% CI -0.87, 4.92, 7 studies) for children 2-5 years. Both reviews did not find any improvement in growth for children. Improved hemoglobin and anemia status could be attributed to the iron component in the MNP. With the benefit on child anemia only, we decided not to add MNP in LiST until further data and evidence on child anemia become available.

Neonatal vitamin A supplementation—neonatal mortality due to diarrhea; child mortality due to diarrhea:

We identified a 2019 IPD analysis on neonatal vitamin A supplementation and infant mortality (78). Only RCTs were included in the review. The intervention was defined as 25,000-50,000 IU vitamin A given within the first 2-3 days of life. And the control group received placebo. The review looked at mortality from data of supplementation to 6 months and to 12 months. All 11 RCTs included in the review were conducted in Asia or Africa.

The reduction in mortality <6 month was not significant in the overall pooled estimates (RR=0.97, 95% CI 0.89-1.06). But in the subgroup analysis, the reduction in mortality <6 months was significant in studies with moderate/severe maternal vitamin A deficiency, defined as greater or equal to 10% (RR=0.87, 95% CI 0.80-0.94, 3 trials). The reduction in mortality <6 months was also significant in studies conducted in Asia (RR=0.87, 95% CI 0.77-0.98, 5 trials). The three trials with moderate/severe maternal vitamin A deficiency were also conducted in Asia.

The review also found that compared to placebo, neonatal vitamin A supplementation did not reduce mortality <12 months (RR=1.00, 95% CI 0.93-1.08, 10 trials). None of the subgroup analysis found a significant impact.

Given the sufficient evidence from the literature, we think it is important to include this intervention. In LiST, we only have cause-specific mortality. The most appropriate cause-specific mortality for neonatal vitamin A supplementation is mortality due to diarrhea for children <6 months. It is noted that the review found 13% reduction on all-cause mortality, our approach might underestimate the actual efficacy of neonatal vitamin A supplementation on mortality due to diarrhea. We define the interventions as percent of neonates receiving 25,000-50,000 IU vitamin A within the first 2-3 days of life. The efficacy is 0.13 and the affected fraction is percent of pregnant women who are vitamin A deficient.

We acknowledge that there are limited data available for percent of pregnant women who are vitamin A deficient. We choose to use prevalence of VAD in pregnant women from the 2009 WHO report. Alternative options that we discussed were using prevalence of night blindness as a proxy or only applying the effect to certain countries with high VAD. Using country as affected fraction is not a common practice in LiST model and we think we are not ready to add this type of affected fraction. Prevalence of night blindness could be a possible proxy as it is one of the symptoms for vitamin A deficiency, however by checking for the country-level prevalence, there were also limited data available for the indicator. Given that the intervention is effective at reducing mortality in settings with high prevalence of VAD, we recommend the use of prevalence of VAD in pregnant women.

We also think this is some good evidence to advocate for vitamin A fortification for mother to improve maternal vitamin A status.

Outcome	Efficacy	Affected fraction
Neonatal mortality due to diarrhea	0.13 (0.06, 0.20)	percent of pregnant women who are vitamin A deficient
Child mortality due to diarrhea	0.13 (0.06, 0.20)	percent of pregnant women who are vitamin A deficient

Neonatal zinc supplementation—neonatal mortality due to sepsis:

We identified a systematic review on zinc for prevention and treatment of sepsis (79). Only RCTs were included in the review. The intervention was oral zinc supplementation in any form and dose with or without other micro-/macronutrient or antibiotics that the control group also received. The control was placebo or a no-zinc intervention. The target population in the review were young infants less than 4 months. There was no restriction on birth weight, gestational ages, and underlying co-morbidities for the included population.

Preventive zinc supplementation for preterm neonates who are at higher risk for sepsis reduced mortality (RR=0.28, 95% CI 0.12-0.67, 2 studies, 265 participants). Evidence from two studies is not sufficient to prove the benefit impact of preventative zinc and mortality among preterm infants. We decided not to add the link between preventive zinc supplementation for preterm neonates and mortality. But given the potential benefits, we think this is an area where more research is needed to better understand the risk association.

For zinc supplementation as a therapeutic intervention for infants with sepsis, the intervention did not reduce all-cause mortality (RR=0.66, 0.40-1.08; 5 studies). In the subgroup analysis by dosage, only dose of 3mg/kg/twice a day was found to be effective at reducing mortality (RR=0.49, 95% CI 0.27-0.87, 2

studies, 359 participants). Evidence from two studies is not sufficient to prove the benefit impact of zinc for treatment of sepsis and mortality. But given the potential benefits, we think this is an area where more research is needed to better understand the risk association.

Section Three: LiST-specific quality checklist

GRADE criteria	Our Indicators	Description	GRADE Rating
Study design	Study design	RCTs; non-RCTs: qRCTs, CBAs, ITSs; or controlled observational studies: cohort, case-control, case series; or mixed?	RCT (starts as high quality); Mix of RCT and non-RCT (start as moderate); Non-RCT (starts as low quality)
Risk of bias	Risk of bias	Any exclusion for studies with lower quality? or did they conduct sensitivity analysis for attrition rate, allocation concealment , high vs. low quality studies?<br>Does the systematic review assess RoB for included studies?	No<br>serious (-1)<br>very serious (-2)
Inconsistency	Heterogeneity	I square statistics<br>0% to 40% might not be important (low heterogeneity)<br>30% to 60% might represent moderate heterogeneity<br>50% to 90% might represent substantial heterogeneity<br>75% to 100% might represent considerable heterogeneity.<br>Wide variation in the effect estimates across studies? Overlap of CIs?	No<br>serious (-1)<br>very serious (-2)
Indirectness	Population	Are the target population clearly defined?	No<br>serious (-1)<br>very serious (-2)
	Study settings	All in LMICs, majority in LMICs, some in LMICs, or none in LMICs?	
	Intervention	Is the intervention clearly defined?	
	Comparator	Is the comparison group appropriate, i.e. a true placebo?	
	Outcomes	Is the outcome what we are interested in, ex cause-specific mortality?	
Imprecision	Number of events/populations	Sufficient if >300 events for dichotomous outcomes, unless event rates are very low<br>Sufficient if >400 participants for continuous outcomes	No<br>serious (-1)<br>very serious (-2)
	Number of studies included	Search studies through which year? How many numbers of studies were included?	
	95% CI	Sufficient if enough participants and the CI is significant, Downgrade if:<br>Dichotomous outcomes--Appreciable benefit or harm is a relative risk reduction or increase greater than 25%, check absolute effects for rare events<br>Continuous outcomes—upper or lower confidence limit crosses effect size (SMD) of 0.5 in either direction	

Publication Bias	Study sizes	If only small studies were included or funnel plot is skewed, publication bias may be present (max to downgrade one level)	Undetected<br>Strongly suspected (-1)
Other (upgrading factors)	RR	Large RR<0.5 or >2 (based on least 2 studies, with no plausible confounder): upgrade 1 level<br>Very large RR<0.2 or >5: upgrade 2 levels	Large effect (+1 or +2)<br>Dose response (+1 or +2)<br>No Plausible confounding (+1 or +2)
	Dose-dependent gradient	Is high dose associated with greater impact?	
	Confounding factors	All plausible confounding from studies may be working against the direction of the observed effect	

Section Four: Meta-analysis for provision of complementary food using loose criteria

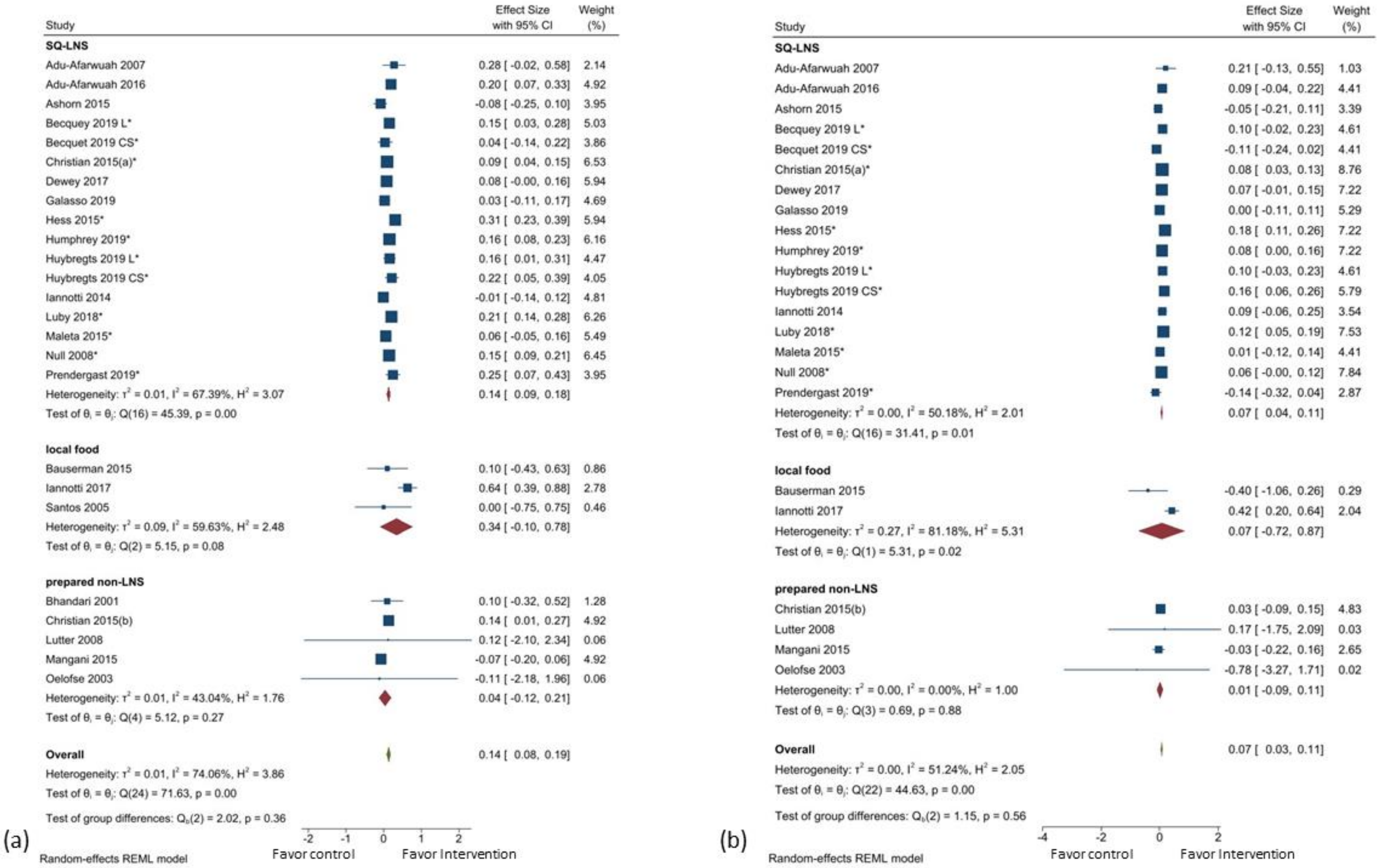

Supplemental Figure 1: Provision of complementary food vs. no supplemental complementary food, (a) mean difference in height-for=age z-score; (b) mean difference in weight-for-height z-score.

Reference

1. Ota E, Hori H, Mori R, Tobe-Gai R, Farrar D. Antenatal dietary education and supplementation to increase energy and protein intake. Cochrane Database Syst Rev. 2015(6):CD000032.
2. Lassi ZS, Padhani ZA, Rabbani A, Rind F, Salam RA, Das JK, et al. Impact of Dietary Interventions during Pregnancy on Maternal, Neonatal, and Child Outcomes in Low- and Middle-Income Countries. Nutrients. 2020;12(2).
3. Huybregts L, Roberfroid D, Lanou H, Menten J, Meda N, Van Camp J, et al. Prenatal food supplementation fortified with multiple micronutrients increases birth length: a randomized controlled trial in rural Burkina Faso. Am J Clin Nutr. 2009;90(6):1593-600.
4. Dwarkanath P, Hsu JW, Tang GJ, Anand P, Thomas T, Thomas A, et al. Energy and Protein Supplementation Does Not Affect Protein and Amino Acid Kinetics or Pregnancy Outcomes in Underweight Indian Women. J Nutr. 2016;146(2):218-26.
5. Jackson BD, Walker N, Heidkamp R. Metrics for Identifying Food Security Status and the Population with Potential to Benefit from Nutrition Interventions in the Lives Saved Tool (LiST). J Nutr. 2017;147(11):2147S-55S.
6. Ceesay SM, Prentice AM, Cole TJ, Foord F, Weaver LT, Poskitt EM, et al. Effects on birth weight and perinatal mortality of maternal dietary supplements in rural Gambia: 5 year randomised controlled trial. Bmj. 1997;315(7111):786-90.
7. Mora J, Navarro L, Clement J, Wagner M, De Paredes B, Herrera MG. The effect of nutritional supplementation on calorie and protein intak eof pregnant women. Nutr Rep Int. 1978:217-28.
8. Prentice AM, Cole TJ, Foord FA, Lamb WH, Whitehead RG. Increased birthweight after prenatal dietary supplementation of rural African women. The American Journal of Clinical Nutrition. 1987;46(6):912-25.
9. Girija A, Geervani P, Rao GN. Influence of Dietary Supplementation During Pregnancy on Lactation Performance. Journal of Tropical Pediatrics. 1984;30(2):79-83.
10. Suite of Food Security Indicators [Available from: https://www.fao.org/faostat/en/#data/FS.
11. Ballard TJ, Kepple AW, Cafiero C. The food insecurity experience scale: developing a global standard for monitoring hunger worldwide. Technical Paper. Rome: FAO; 2013.
12. Imdad A, Jabeen A, Bhutta ZA. Role of calcium supplementation during pregnancy in reducing risk of developing gestational hypertensive disorders: a meta-analysis of studies from developing countries. BMC Public Health. 2011;11 Suppl 3:S18.
13. Hofmeyr GJ, Lawrie TA, Atallah AN, Torloni MR. Calcium supplementation during pregnancy for preventing hypertensive disorders and related problems. Cochrane Database Syst Rev. 2018;10:CD001059.
14. Oh C, Keats EC, Bhutta ZA. Vitamin and Mineral Supplementation During Pregnancy on Maternal, Birth, Child Health and Development Outcomes in Low- and Middle-Income Countries: A Systematic Review and Meta-Analysis. Nutrients. 2020;12(2).
15. Beal T, Massiot E, Arsenault JE, Smith MR, Hijmans RJ. Global trends in dietary micronutrient supplies and estimated prevalence of inadequate intakes. PLoS One. 2017;12(4):e0175554.
16. Villar J, Abdel-Aleem H, Merialdi M, Mathai M, Ali MM, Zavaleta N, et al. World Health Organization randomized trial of calcium supplementation among low calcium intake pregnant women. Am J Obstet Gynecol. 2006;194(3):639-49.
17. Pena-Rosas JP, De-Regil LM, Garcia-Casal MN, Dowswell T. Daily oral iron supplementation during pregnancy. Cochrane Database Syst Rev. 2015(7):CD004736.
18. Keats EC, Haider BA, Tam E, Bhutta ZA. Multiple-micronutrient supplementation for women during pregnancy. Cochrane Database Syst Rev. 2019;3:CD004905.

19. Haider BA, Olofin I, Wang M, Spiegelman D, Ezzati M, Fawzi WW, et al. Anaemia, prenatal iron use, and risk of adverse pregnancy outcomes: systematic review and meta-analysis. BMJ. 2013;346:f3443.
20. Smith ER, Shankar AH, Wu LSF, Aboud S, Adu-Afarwuah S, Ali H, et al. Modifiers of the effect of maternal multiple micronutrient supplementation on stillbirth, birth outcomes, and infant mortality: a meta-analysis of individual patient data from 17 randomised trials in low-income and middle-income countries. The Lancet Global Health. 2017;5(11):e1090-e100.
21. Christian P, Khatry SK, Katz J, Pradhan EK, LeClerq SC, Shrestha SR, et al. Effects of alternative maternal micronutrient supplements on low birth weight in rural Nepal: double blind randomised community trial. BMJ (Clinical research ed). 2003;326(7389):571.
22. Keats EC, Neufeld LM, Garrett GS, Mbuya MNN, Bhutta ZA. Improved micronutrient status and health outcomes in low- and middle-income countries following large-scale fortification: evidence from a systematic review and meta-analysis. Am J Clin Nutr. 2019;109(6):1696-708.
23. Tong H, Walker N. Current levels of coverage of iron and folic acid fortification are insufficient to meet the recommended intake for women of reproductive age in low- and middle-income countries. J Glob Health. 2021;11:18002.
24. Imdad A, Yakoob MY, Bhutta ZA. The effect of folic acid, protein energy and multiple micronutrient supplements in pregnancy on stillbirths. BMC Public Health. 2011;11 Suppl 3(Suppl 3):S4.
25. Li B, Zhang X, Peng X, Zhang S, Wang X, Zhu C. Folic Acid and Risk of Preterm Birth: A Meta-Analysis. Front Neurosci. 2019;13:1284.
26. Li Z, Ye R, Zhang L, Li H, Liu J, Ren A. Periconceptional folic acid supplementation and the risk of preterm births in China: a large prospective cohort study. Int J Epidemiol. 2014;43(4):1132-9.
27. Vahratian A, Siega-Riz AM, Savitz DA, Thorp JM, Jr. Multivitamin use and the risk of preterm birth. Am J Epidemiol. 2004;160(9):886-92.
28. Martinussen MP, Bracken MB, Triche EW, Jacobsen GW, Risnes KR. Folic acid supplementation in early pregnancy and the risk of preeclampsia, small for gestational age offspring and preterm delivery. Eur J Obstet Gynecol Reprod Biol. 2015;195:94-9.
29. Zeitlin J, Szamotulska K, Drewniak N, Mohangoo AD, Chalmers J, Sakkeus L, et al. Preterm birth time trends in Europe: a study of 19 countries. BJOG. 2013;120(11):1356-65.
30. Blencowe H, Kancherla V, Moorthie S, Darlison MW, Modell B. Estimates of global and regional prevalence of neural tube defects for 2015: a systematic analysis. Ann N Y Acad Sci. 2018;1414(1):31-46.
31. Palacios C, Kostiuk LK, Pena-Rosas JP. Vitamin D supplementation for women during pregnancy. Cochrane Database Syst Rev. 2019;7:CD008873.
32. Chamberlain C, O'Mara-Eves A, Porter J, Coleman T, Perlen SM, Thomas J, et al. Psychosocial interventions for supporting women to stop smoking in pregnancy. Cochrane Database Syst Rev. 2017;2:CD001055.
33. Salam RA, Das JK, Bhutta ZA. Effect of mass deworming with antihelminthics for soil-transmitted helminths during pregnancy. Cochrane Database Syst Rev. 2021;5:CD005547.
34. Smith TJ, Hess SY. Infantile thiamine deficiency in South and Southeast Asia: An age-old problem needing new solutions. Nutr Bull. 2021;46(1):12-25.
35. Tsang BL, Holsted E, McDonald CM, Brown KH, Black R, Mbuya MNN, et al. Effects of Foods Fortified with Zinc, Alone or Cofortified with Multiple Micronutrients, on Health and Functional Outcomes: A Systematic Review and Meta-Analysis. Adv Nutr. 2021;12(5):1821-37.
36. Carducci B, Keats EC, Bhutta ZA. Zinc supplementation for improving pregnancy and infant outcome. Cochrane Database Syst Rev. 2021;3:CD000230.
37. Ota E, Mori R, Middleton P, Tobe-Gai R, Mahomed K, Miyazaki C, et al. Zinc supplementation for improving pregnancy and infant outcome. Cochrane Database Syst Rev. 2015(2):CD000230.

38. Middleton P, Gomersall JC, Gould JF, Shepherd E, Olsen SF, Makrides M. Omega-3 fatty acid addition during pregnancy. Cochrane Database Syst Rev. 2018;11:CD003402.
39. Sinha B, Chowdhury R, Upadhyay RP, Taneja S, Martines J, Bahl R, et al. Integrated Interventions Delivered in Health Systems, Home, and Community Have the Highest Impact on Breastfeeding Outcomes in Low- and Middle-Income Countries. J Nutr. 2017;147(11):2179S-87S.
40. Lassi ZS, Rind F, Irfan O, Hadi R, Das JK, Bhutta ZA. Impact of Infant and Young Child Feeding (IYCF) Nutrition Interventions on Breastfeeding Practices, Growth and Mortality in Low- and Middle-Income Countries: Systematic Review. Nutrients. 2020;12(3).
41. Walker CL, Black RE. Zinc for the treatment of diarrhoea: effect on diarrhoea morbidity, mortality and incidence of future episodes. Int J Epidemiol. 2010;39 Suppl 1:i63-9.
42. Lazzerini M, Wanzira H. Oral zinc for treating diarrhoea in children. Cochrane Database Syst Rev. 2016;12:CD005436.
43. Panjwani A, Heidkamp R. Complementary Feeding Interventions Have a Small but Significant Impact on Linear and Ponderal Growth of Children in Low- and Middle-Income Countries: A Systematic Review and Meta-Analysis. J Nutr. 2017;147(11):2169S-78S.
44. Dewey KG, Wessells KR, Arnold CD, Prado EL, Abbeddou S, Adu-Afarwuah S, et al. Characteristics that modify the effect of small-quantity lipid-based nutrient supplementation on child growth: an individual participant data meta-analysis of randomized controlled trials. Am J Clin Nutr. 2021;114(Suppl 1):15S-42S.
45. Maleta KM, Phuka J, Alho L, Cheung YB, Dewey KG, Ashorn U, et al. Provision of 10-40 g/d Lipid-Based Nutrient Supplements from 6 to 18 Months of Age Does Not Prevent Linear Growth Faltering in Malawi. J Nutr. 2015;145(8):1909-15.
46. Mangani C, Maleta K, Phuka J, Cheung YB, Thakwalakwa C, Dewey K, et al. Effect of complementary feeding with lipid-based nutrient supplements and corn-soy blend on the incidence of stunting and linear growth among 6- to 18-month-old infants and children in rural Malawi. Matern Child Nutr. 2015;11 Suppl 4:132-43.
47. Olaya GA, Lawson M, Fewtrell MS. Efficacy and safety of new complementary feeding guidelines with an emphasis on red meat consumption: a randomized trial in Bogota, Colombia. Am J Clin Nutr. 2013;98(4):983-93.
48. Stephenson KB, Agapova SE, Divala O, Kaimila Y, Maleta KM, Thakwalakwa C, et al. Complementary feeding with cowpea reduces growth faltering in rural Malawian infants: a blind, randomized controlled clinical trial. Am J Clin Nutr. 2017;106(6):1500-7.
49. Lutter CK, Rodríguez A, Fuenmayor G, Avila L, Sempertegui F, Escobar J. Growth and micronutrient status in children receiving a fortified complementary food. J Nutr. 2008;138(2):379-88.
50. Oelofse A, Van Raaij JM, Benade AJ, Dhansay MA, Tolboom JJ, Hautvast JG. The effect of a micronutrient-fortified complementary food on micronutrient status, growth and development of 6- to 12-month-old disadvantaged urban South African infants. Int J Food Sci Nutr. 2003;54(5):399-407.
51. Stewart CP, Wessells KR, Arnold CD, Huybregts L, Ashorn P, Becquey E, et al. Lipid-based nutrient supplements and all-cause mortality in children 6-24 months of age: a meta-analysis of randomized controlled trials. Am J Clin Nutr. 2020;111(1):207-18.
52. Wessells KR, Arnold CD, Stewart CP, Prado EL, Abbeddou S, Adu-Afarwuah S, et al. Characteristics that modify the effect of small-quantity lipid-based nutrient supplementation on child anemia and micronutrient status: an individual participant data meta-analysis of randomized controlled trials. Am J Clin Nutr. 2021;114(Suppl 1):68S-94S.
53. Black RE, Victora CG, Walker SP, Bhutta ZA, Christian P, de Onis M, et al. Maternal and child undernutrition and overweight in low-income and middle-income countries. The Lancet. 2013;382(9890):427-51.

54. Imdad A, Mayo-Wilson E, Herzer K, Bhutta ZA. Vitamin A supplementation for preventing morbidity and mortality in children from six months to five years of age. Cochrane Database Syst Rev. 2017;3:CD008524.
55. Tam E, Keats EC, Rind F, Das JK, Bhutta AZA. Micronutrient Supplementation and Fortification Interventions on Health and Development Outcomes among Children Under-Five in Low- and Middle-Income Countries: A Systematic Review and Meta-Analysis. Nutrients. 2020;12(2).
56. Fisker AB, Bale C, Rodrigues A, Balde I, Fernandes M, Jørgensen MJ, et al. High-dose vitamin A with vaccination after 6 months of age: a randomized trial. Pediatrics. 2014;134(3):e739-48.
57. Chen K, Chen XR, Zhang L, Luo HY, Gao N, Wang J, et al. Effect of simultaneous supplementation of vitamin A and iron on diarrheal and respiratory tract infection in preschool children in Chengdu City, China. Nutrition. 2013;29(10):1197-203.
58. Imdad A, Yakoob MY, Sudfeld C, Haider BA, Black RE, Bhutta ZA. Impact of vitamin A supplementation on infant and childhood mortality. BMC Public Health. 2011;11 Suppl 3:S20.
59. Awasthi S, Peto R, Read S, Richards SM, Pande V, Bundy D. Population deworming every 6 months with albendazole in 1 million pre-school children in North India: DEVTA, a cluster-randomised trial. Lancet. 2013;381(9876):1478-86.
60. West KP, Jr., Sommer A, Palmer A, Schultink W, Habicht JP. Commentary: Vitamin A policies need rethinking. Int J Epidemiol. 2015;44(1):292-4; discussion 4-6.
61. Sommer A, West KP, Martorell R. Vitamin A supplementation in Indian children. The Lancet. 2013;382(9892).
62. Habicht JP, Pelto GH. From biological to program efficacy: promoting dialogue among the research, policy, and program communities. Adv Nutr. 2014;5(1):27-34.
63. Stevens GA, Bennett JE, Hennocq Q, Lu Y, De-Regil LM, Rogers L, et al. Trends and mortality effects of vitamin A deficiency in children in 138 low-income and middle-income countries between 1991 and 2013: a pooled analysis of population-based surveys. The Lancet Global Health. 2015;3(9):e528-e36.
64. Yakoob MY, Theodoratou E, Jabeen A, Imdad A, Eisele TP, Ferguson J, et al. Preventive zinc supplementation in developing countries: impact on mortality and morbidity due to diarrhea, pneumonia and malaria. BMC Public Health. 2011;11 Suppl 3:S23.
65. Brown KH, Peerson JM, Baker SK, Hess SY. Preventive zinc supplementation among infants, preschoolers, and older prepubertal children. Food Nutr Bull. 2009;30(1 Suppl):S12-40.
66. Bhutta ZA, Das JK, Rizvi A, Gaffey MF, Walker N, Horton S, et al. Evidence-based interventions for improvement of maternal and child nutrition: what can be done and at what cost? Lancet. 2013;382(9890):452-77.
67. Mayo-Wilson E, Junior JA, Imdad A, Dean S, Chan XH, Chan ES, et al. Zinc supplementation for preventing mortality, morbidity, and growth failure in children aged 6 months to 12 years of age. Cochrane Database Syst Rev. 2014(5):CD009384.
68. Wessells KR, Brown KH. Estimating the global prevalence of zinc deficiency: results based on zinc availability in national food supplies and the prevalence of stunting. PLoS One. 2012;7(11):e50568.
69. Hess SY. National Risk of Zinc Deficiency as Estimated by National Surveys. Food Nutr Bull. 2017;38(1):3-17.
70. Thayer WM, Clermont A, Walker N. Effects of deworming on child and maternal health: a literature review and meta-analysis. BMC Public Health. 2017;17(Suppl 4):830.
71. Taylor-Robinson DC, Maayan N, Soares-Weiser K, Donegan S, Garner P. Deworming drugs for soil-transmitted intestinal worms in children: effects on nutritional indicators, haemoglobin, and school performance. Cochrane Database Syst Rev. 2015(7):CD000371.
72. Croke K, Hicks J, Hsu E, Kremer M, Miguel E. Does Mass Deworming Affect Child Nutrition? Meta-analysis, Cost-Effectiveness, and Statistical Power. National Bureau of Economic Research 2016.

73. Martineau AR, Jolliffe DA, Hooper RL, Greenberg L, Aloia JF, Bergman P, et al. Vitamin D supplementation to prevent acute respiratory tract infections: systematic review and meta-analysis of individual participant data. BMJ. 2017;356:i6583.
74. Huey SL, Acharya N, Silver A, Sheni R, Yu EA, Pena-Rosas JP, et al. Effects of oral vitamin D supplementation on linear growth and other health outcomes among children under five years of age. Cochrane Database Syst Rev. 2020;12:CD012875.
75. Margolies A, Kemp CG, Choo EM, Levin C, Olney D, Kumar N, et al. Nutrition-sensitive agriculture programs increase dietary diversity in children under 5 years: A review and meta-analysis. J Glob Health. 2022;12:08001.
76. Suchdev PS, Jefferds MED, Ota E, da Silva Lopes K, De-Regil LM. Home fortification of foods with multiple micronutrient powders for health and nutrition in children under two years of age. Cochrane Database Syst Rev. 2020;2:CD008959.
77. De-Regil LM, Jefferds MED, Pena-Rosas JP. Point-of-use fortification of foods with micronutrient powders containing iron in children of preschool and school-age. Cochrane Database Syst Rev. 2017;11:CD009666.
78. Neonatal Vitamin ASEg. Early neonatal vitamin A supplementation and infant mortality: an individual participant data meta-analysis of randomised controlled trials. Arch Dis Child. 2019;104(3):217-26.
79. Irfan O, Black RE, Lassi ZS, Bhutta ZA. Zinc Supplementation and the Prevention and Treatment of Sepsis in Young Infants: A Systematic Review and Meta-Analysis. Neonatology. 2022:1-12.
